# Single-cell transcriptome reveals core cell populations and androgen-*RXFP2* axis involved in deer antler full regeneration

**DOI:** 10.1186/s13619-022-00153-4

**Published:** 2022-12-21

**Authors:** Hengxing Ba, Xin Wang, Datao Wang, Jing Ren, Zhen Wang, Hai-Xi Sun, Pengfei Hu, Guokun Zhang, Shengnan Wang, Chao Ma, Yusu Wang, Enpeng Wang, Liang Chen, Tianbin Liu, Ying Gu, Chunyi Li

**Affiliations:** 1 Institute of Antler Science and Product Technology, Changchun Sci-Tech University, Changchun, 130600 China; 2 Jilin Provincial Key Laboratory of Deer Antler Biology, Changchun, 130600 China; 3 BGI-Shenzhen, Shenzhen, 518083 Guangdong China; 4 Hubei Key Laboratory of Cell Homeostasis, College of Life Sciences, RNA Institute, Wuhan University, Wuhan, China; 5 Institute of Special Wild Economic Animals and Plants, Chinese Academy of Agricultural Sciences, 130112, Changchun, China; 6 Jilin Ginseng Academy, Changchun University of Chinese Medicine, Changchun, 130117 China; 7 College of Life Sciences, University of Chinese Academy of Sciences, Beijing, 100049 China; 8 Guangdong Provincial Key Laboratory of Genome Read and Write, BGI-Shenzhen, Shenzhen, 518120 Guangdong China; 9 College of Chinese Medicinal Materials, Jilin Agricultural University, Changchun, 130118 China

**Keywords:** Antler, *THY1*^+^ cell, Generation, Regeneration, Core cell population, Androgen-RXFP2 axis, Single cell sequencing

## Abstract

Deer antlers constitute a unique mammalian model for the study of both organ formation in postnatal life and annual full regeneration. Previous studies revealed that these events are achieved through the proliferation and differentiation of antlerogenic periosteum (AP) cells and pedicle periosteum (PP) cells, respectively. As the cells resident in the AP and the PP possess stem cell attributes, both antler generation and regeneration are stem cell-based processes. However, the cell composition of each tissue type and molecular events underlying antler development remain poorly characterized. Here, we took the approach of single-cell RNA sequencing (scRNA-Seq) and identified eight cell types (mainly *THY1*^+^ cells, progenitor cells, and osteochondroblasts) and three core subclusters of the *THY1*^+^ cells (SC2, SC3, and SC4). Endothelial and mural cells each are heterogeneous at transcriptional level. It was the proliferation of progenitor, mural, and endothelial cells in the activated antler-lineage-specific tissues that drove the rapid formation of the antler. We detected the differences in the initial differentiation process between antler generation and regeneration using pseudotime trajectory analysis. These may be due to the difference in the degree of stemness of the AP-THY1^+^ and PP-THY1^+^ cells. We further found that androgen-RXFP2 axis may be involved in triggering initial antler full regeneration. Fully deciphering the cell composition for these antler tissue types will open up new avenues for elucidating the mechanism underlying antler full renewal in specific and regenerative medicine in general.

**Supplementary Information:**

The online version contains supplementary material available at 10.1186/s13619-022-00153-4.

## Background

Regeneration of lost organs/appendages is the “Holy Grail” of regenerative medicine, known as “epimorphic regeneration” (Stocum 2002). The established animal models in this area include mouse digit tips, planarians, zebrafish, and newts. Our current knowledge of epimorphic regeneration is mainly built up through the investigation of these model animals. Collectively, it is essential for enabling epimorphic regeneration to form a cone-shaped cell mass, known as a blastema, firstly on the amputed plane of an appendage stump through either cell dedifferentiation process (Mescher 1996), residential stem cell activation (Weissman et al. 2001) or cell transdifferentiation (Jopling et al. 2011). However, thus far, the natural mammalian model of epimorphic regeneration has not been entirely absent but very scarce. Surprisingly, deer antlers, a large complex mammalian organ, can not only fully regenerate once they fall off from their pedicles (permanent bony protuberances) but also do so annually. Thus they can serve as a unique model to explore how nature has solved the problem of mammalian epimorphic regeneration (Goss 1983). Deer are not born with pedicles and antlers. As a male secondary sexual character, pedicles start to grow when male deer approach puberty and circulating androgen hormones rapidly increase (Li et al. 2003; Suttie et al. 1991), and the first antler spontaneously transforms from the pedicles when they reach a species-specific height (such as sika deer, around 5 cm; (Li and Suttie 1994b).

Previous studies have demonstrated that the potential to form a pedicle and first antler (antler generation) resides in the periosteum overlying the presumptive pedicle growth region; this is termed the antlerogenic periosteum (AP; Fig. S1A). Deletion of the AP prevents the development of pedicles and subsequent antlers, but autologous transplantation of the AP elsewhere induces ectopic or even xenogeneic pedicle and antler formation in nude mice (Fig. S1B) (Goss 1987, 1990; Landete-Castillejos et al. 2019). Through a likewise approach, Li et al. (2007) successfully identified the tissue type that gives rise to a regenerating antler, namely the pedicle periosteum (PP; Fig. S1C). Deletion of the PP abrogates antler regeneration (Fig. S1D) (Li et al. 2007). Initiation of antler regeneration starts with the formation of a blastema (Fig. S1E) through activation of distal PP cells (Li and Chu 2016), and the histological makeup of the blastema (Fig. S1F) is essentially reminiscent of the growth center of the early growing antler (Li et al. 2005). Cells of both AP and PP possess stem cell attributes, such as express pluripotent/mesenchymal stem cell markers, in vitro can be induced to differentiate into multiple cell lineages and self-renew (Li et al. 2009b; Wang et al. 2019). Therefore, antler generation and annual full regeneration are all stem cell-based processes.

Single-cell RNA sequencing (scRNA-seq) techniques can quantify intra-population heterogeneity and enable the learning of cell states and transitions at very high resolution, potentially revealing cell subtypes or gene expression dynamics (Liu and Trapnell 2016). In terms of epimorphic regeneration, scRNA-seq techniques have been used to define the cell composition of the blastema in axolotl and to reveal the mechanism underlying dedifferentiation-based regeneration (Gerber et al. 2018; Leigh et al. 2018; Li et al. 2021). However, the equivalent investigation for stem cell-based regeneration needs to be improved. In the present study, we took the scRNA-seq approach and sequenced AP and PP (antler lineage tissues, AnLTs) to determine 1) whether the niche of each tissue AnLT contains a homogeneous or heterogeneous cell population; 2) if heterogeneous, was there a dominant subpopulation responsible for the assigned particular function, or was the outcome a function of a collective endeavor? 3) molecular events initiating antler generation and regeneration. It is necessary to clearly address these questions if successful strategies are to be devised for the stem cell-based restoration/regeneration of damaged/lost organs in mammals, particularly in humans.

## Results

### Cell composition of each tissue type in AnLTs

**Fig. 1:**
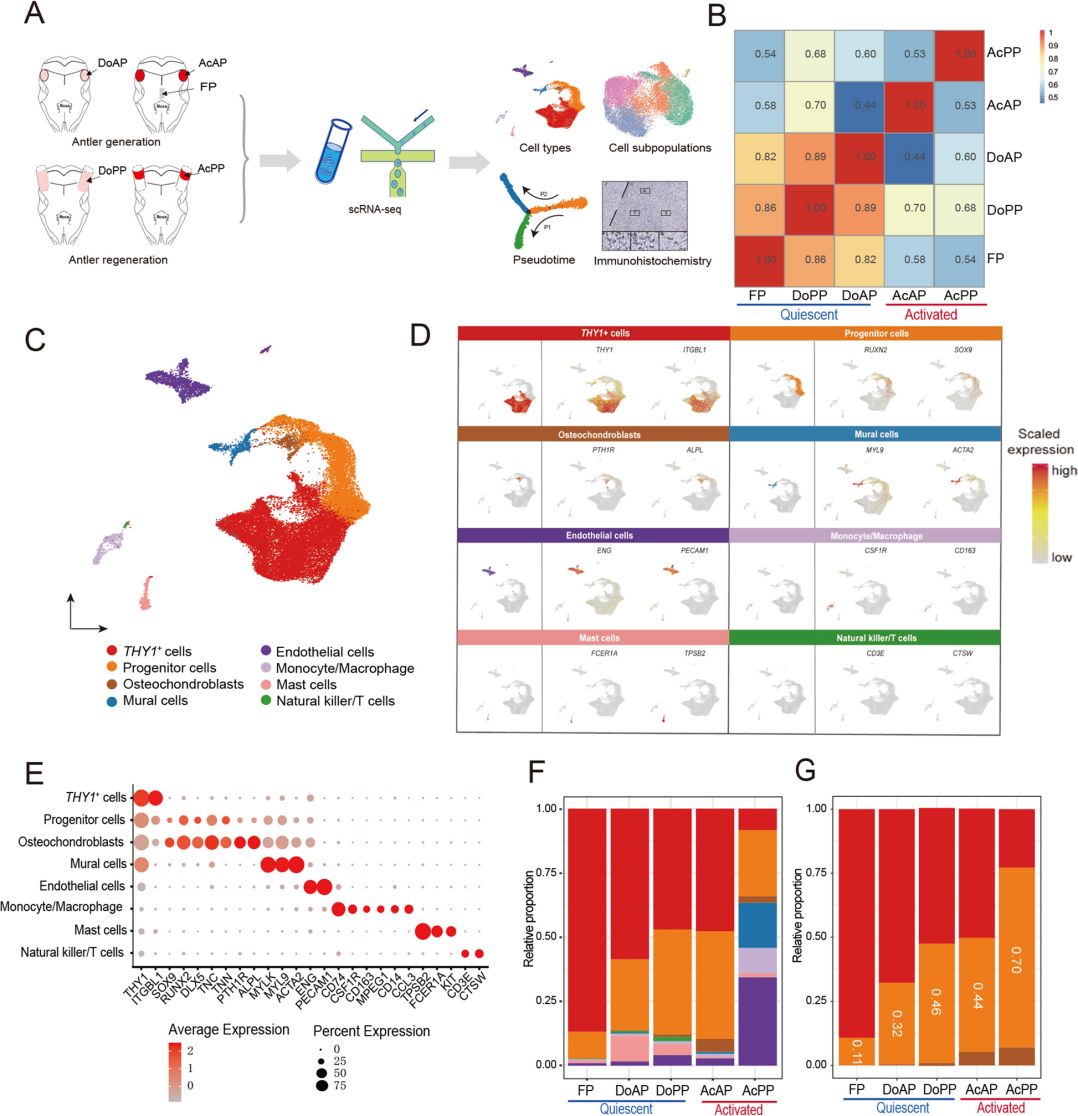
Single-cell profiling of AnLTs and FP. **A** Schematic drawing of the study design and workflow. Three separate samples of each tissue type were pooled. DoAP, dormant antlerogenic periosteum; AcAP, activated antlerogenic periosteum; DoPP, dormant pedicle periosteum; AcPP, activated pedicle periosteum; FP, facial periosteum. **B** Heatmap of correlations between different tissue types, calculated based on scRNA-seq data. Pearson correlation coefficients were shown. **C** UMAP to visualize all cells in the five tissue types, colored to distinguish cell types of the tissue samples. Cell types were identified based on the expression status of known marker genes. **D** UMAP to show the expression patterns of marker genes in each cell type. The left panel: the distribution of each cell type; the middle and the right panels: the expression levels of the representative marker genes in each cell type. **E** Dot plot to show the expression status of marker genes in each cell type. The size of the dot represents the percentage of cells expressing this marker gene in this cell type, and the depth of color represents the average expression level of this marker gene in this cell type. **F** Bar plot of the relative proportion of each cell type in the five tissue types. Different colors represent different cell types, refer to (**C**). **G** Bar plot of relative proportions of three types of the bone-lineage cells (*THY1*^+^ cells, progenitor cells, and osteochondroblasts) in each tissue type. The color scheme is consistent with (**C**)

We collected four types of AnLTs to perform scRNA-seq, including dormant AP (DoAP), activated AP (AcAP), dormant PP (DoPP), and activated PP (AcPP, equivalent to the reserve mesenchymal layer of an early antler growth center), and using deer facial periosteum (FP, a tissue type that is relatively quiescent and non-regenerative) as a control tissue (Fig. 1A). Both AcAP and AcPP are mitotically-active and termed activated AnLTs; in contrast, DoAP and DoPP are mitotically-quiescent and termed quiescent AnLTs.

We integrated the resultant scRNA-seq data from these different tissue samples. In total, >28,000 cells were obtained after rigorous quality control filtering (Table S1). The global comparison of the transcriptomes between these tissue samples showed that there were higher correlations between the quiescent AnLTs and FP, whereas there was a lower degree of correlation between the activated AnLTs and the quiescent periosteal tissues (Fig. 1B). This highlights the major changes occurred from quiescence to activation for the AnLTs during antler ontogeny, which is consistent with the data from our bulk RNA-seq data analysis (Fig. S2A).

Using unsupervised clustering analysis, we identified eight cell types (*THY1*^+^ cells, progenitor cells, osteochondroblasts, mural cells, endothelial cells, monocytes/macrophages, mast cells, and natural killer/T cells) from the five sequenced tissue samples based on their corresponding marker genes (Figs. 1C, D, E; Figs. S2B, C, D, E and Table S2). Among these cell types, *THY1*^+^ cells had been defined in our previous studies (Ba et al. 2019a; Dong et al. 2020; Wang et al. 2019); progenitor cells were defined for their higher expression of *RUNX2* (Prince et al. 2001), *PTN* (Dong et al. 2021), *SOX9* (Kim and Im 2011), *DLX5* (Yang et al. 2020), *TNN* (Lui et al. 2015) and *TNC* (Liu et al. 2019), prior to differentiation into osteochondroblasts. *SOX9* and *RUNX2* are essential for the differentiation of mesenchymal stem cell-derived osteochondro-progenitors towards chondrogenesis and osteogenesis, respectively, but *SOX9* is dominant over *RUNX2* function in mesenchymal precursors that are destined for a chondrogenic lineage during endochondral ossification (Zhou et al. 2006).

Based on these analyses, we found that majority of the identified cell types were present in all tissue samples, including both AnLTs and FP; some of the cell types, however, were tissue-specific, such as endothelial cells and mural cells, which were more abundant in the AcPP (Fig. 1F), suggesting the AcPP tissue has rich supply of blood vessels, possibly associated with the needs of rapid formation of antler growth center, and consistent with our previous anatomical characterization of AcPP (Li et al. 2002). Generally, the more differentiated the tissue, the smaller the proportion of *THY1*^+^ cells and the larger proportion of progenitor cells (Fig. 1G). These observations indicate that antler growth can be viewed as a process of gradual decline in *THY1*^+^ cell number and increase in progenitor cells. The lowest proportion of progenitor cells was found in the non-antlerogenic tissue (FP), possibly because FP is a type of more mitotically-quiescent and non-regenerative tissue compared to other AnLTs. Our results demonstrate a dynamic change in cell composition in the niches of each type AnLT during both antler generation and regeneration; further, the tissue-specific characteristics of each AnLTs may be reflected by their specific cell composition (as compared with the non-antlerogenic tissue).

### Cell cycling cells were mainly the progenitor, mural, and endothelial cells in the activated AnLTs

**Fig. 2:**
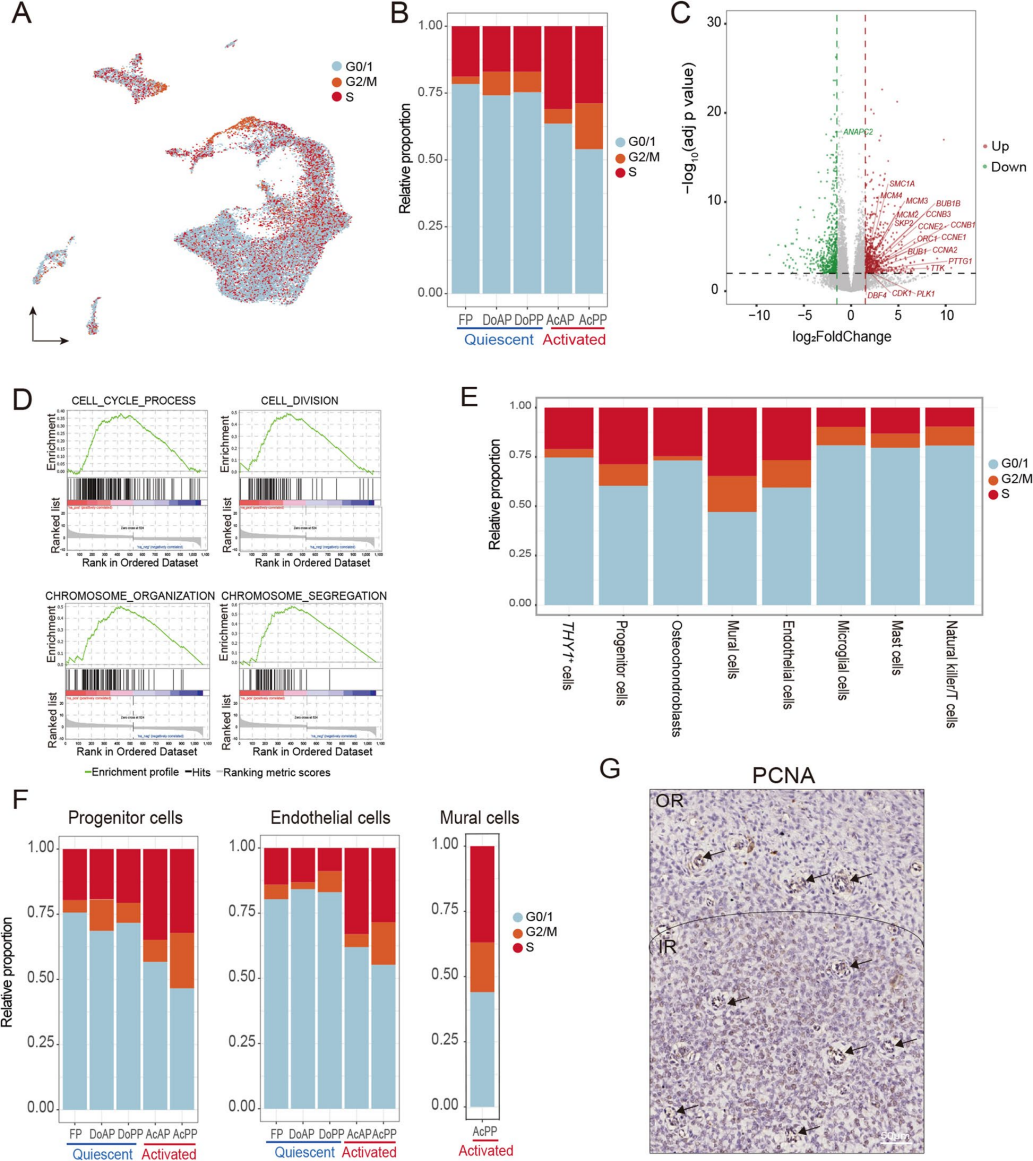
Progenitor, mural, and endothelial cells mainly labeled as proliferative cells. **A** UMAP to show the distribution of the different cell cycle phases. **B** Bar plot to show the proportion of different cell cycle phases in different tissue types. **C** Volcano plot to show the DEGs of bulk RNA-seq between the activated AnLTs (AcAP and AcPP) and the mitotically-quiescent periosteal tissues (DoAP, DoPP and FP). Genes found only in the KEGG cell cycle pathway were labeled. **D** GSEA plot to show the cell cycle pathways (gene size >= 15 and nominal *p*-value < 0.005) enriched in the activated AnLTs compared to the mitotically-quiescent tissues. This GSEA enrichment analysis was performed on bulk RNA-seq. **E** Bar plot to show the proportions of different cell cycle phases of each of the eight cell types in the mixture of all five tissue samples. **F** Bar plots to show the proportions of different cell cycle phases of each of the three cell types (progenitor cells, endothelial cells, and mural cells) in five individual tissue samples. For mural cells, the proportion of cell cycle phases is shown only in the AcPP, probably because only a few mural cells are resident in the other tissue types. **G** IHC staining of PCNA in AcPP. Numerous positively stained cells (brown) are shown in the IR; and in the vascular regions of both OR and IR (black arrows). OR, outer sublayer; IR, inner sublayers

Deer antlers have been claimed as the fastest-growing animal tissue, and their growth rate can reach 2 cm/day (Goss 1983). The pedicle and antler formation is achieved through the proliferation and differentiation of different cell types of AnLTs (Li and Suttie 1994b). In the present study, we found that the percentage of mitotically-active cells was significantly higher in the activated AnLTs than that for the mitotically-quiescent tissues (Figs. 2A, B; Fig. S3A). The results analyzed using our bulk RNA-seq data also showed that cell cycle genes were highly overexpressed (Fig. 2C and Table S3), and cell cycle-related terms of gene ontology were significantly enriched in the activated AnLTs (Fig. 2D). The proliferating cells were predominantly the progenitor, mural and endothelial cells (Fig. 2E), which were mainly found in the activated AnLTs, especially in the AcPP (Fig. 2F). Our immunohistochemistry (IHC) staining of PCNA (Fig. 2G) demonstrated that the AcPP tissue layer could be divided into two sublayers: the outer and inner sublayers. The outer sublayer was mitotically-quiescent, whereas the inner sublayer contained intensively dividing cells.

### Characterization of the *THY1*^+^ cells

**Fig. 3:**
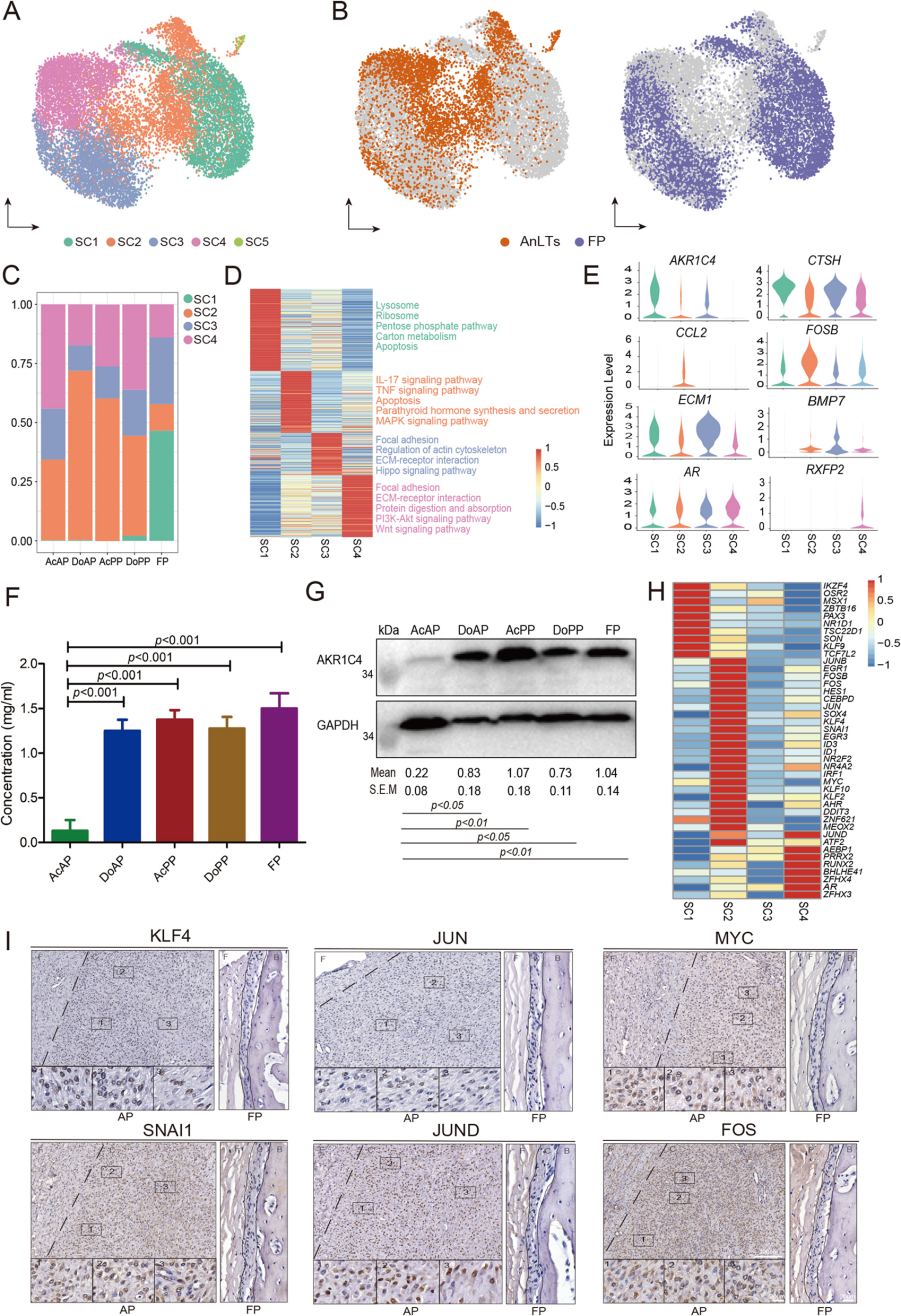
*THY1*^+^ cell subclusters in AnLTs and FP. **A** UMAP plot to visualize the five subclusters (SC1, SC2, SC3, SC4 and SC5) of *THY1*^+^ cells. **B** UMAP plot to visualize the distribution of *THY1*^+^ cell subclusters in the AnLTs and FP, respectively. The left panel: *THY1*^+^ cells of the AnLTs in orange, and the right panel: *THY1*^+^ cells of the FP in purple. **C** Bar plot to show the proportion of each *THY1*^+^ cell subcluster in the five tissue types. **D** Heatmap plot to show the average expression levels of the DEGs in the four *THY1*^+^ cell subclusters (SC1-SC4). The descriptions right to the heatmap are the top KEGG enriched pathways (adjust *p* value < 0.05). **E** Violin plots to show the expression levels of eight key genes in the four *THY1*^+^ cell subclusters (SC1-SC4). **F** Enzymatic activities of AKR1C4 of the five tissue types, were determined via enzyme-linked immunosorbent assay. Value: mean ± S.E.M. *n*=3. **G** Immunoblots of AKR1C4 protein of the five tissue types and internal reference protein (GAPDH). Relative intensity of each immunoblot was calculated using Image J. Value: mean ± S.E.M. *n*=3. **H** Heatmap plot to show the expression levels of the differentially transcriptional factors in the four *THY1*^+^ cell subclusters. **I** IHC staining of six key transcriptional factors in the AP and FP tissues, respectively. Note that numerous positively stained cells (brown) were found in the AP, but not in the FP. AP, antlerogenic periosteum; FP, facial periosteum; F, fibrous layer; C, cellular layer; B, bone

Pedicles and antlers are AnLT-derived (Li et al. 2009b), and the AnLTs must be different in some aspects from those in the FP, contributing to their different differentiation destinies. Some cells in the *THY1*^+^ cell population highly expressed pluripotent stem cell markers *KLF4* and *MYC* (Fig. S3B) that are also the key factors for cell reprogramming (Takahashi and Yamanaka 2006). Thus this population was further divided into subclusters (in total five: SC1, SC2, SC3, SC4, and SC5) via an unsupervised clustering analysis (Fig. 3A and Table S4). Amongst these subclusters, SC2, SC3 and SC4 were more highly correlated, whilst SC1 showed a lower correlation with the other three (Fig. S3C). The SC5 showed the lowest correlation with the rest and was characterized by highly expressed immune-related markers (*CD38*) (Fig. S3D) (Picozza et al. 2013), indicating that it might be of blood origin and therefore was excluded from the subsequent analysis.

Due to the fact that SC1 was specifically found in FP (Figs. 3A, B, C), it is to be expected that this subcluster would not be related to the antlerogenic function. Furthermore, genes involved in the function of the lysosome, the ribosome, and pentose phosphate pathway were found to be highly expressed in SC1 compared to the other subclusters (Fig. 3D and Table S5). Interestingly, the *AKR1C4* gene (Aldo-Keto Reductase Family 1 Member C4) exhibited the highest level of expression in SC1, the intermediate in SC2 and SC3 and the lowest in SC4 (Fig. 3E). The result that the FP exhibited the highest expression level of AKR1C4 protein was confirmed using ELISA quantitative (Fig. 3F) and Western blotting analyses (Fig. 3G): FP had the strongest enzyme activity (≈1.49 mg/ml); DoAP, DoPP and AcPP had medium (≈1.27 mg/ml), but AcAP had the lowest (≈0.15 mg/ml). It is reported that *AKR1C4* gene encodes an enzyme that can attenuate androgen potency (Penning et al. 2000; Steckelbroeck et al. 2004). These results indicated that SC1 may mediate a process of attenuating the potency of androgen hormone signals to ensure that the non-antlerogenic tissues (FP) are unable to respond to the type and level of androgens that would otherwise stimulate the DoAP to initiate pedicle formation.

The SC2 subcluster was distributed mainly in the AnLTs with a negligible number in the FP (Fig. 3C); hence, it was annotated as an AnLT-specific subcluster. Further analysis showed that SC2 exhibited high levels of expression of some transcriptional factors, including those required for the maintenance of stemness (*KLF4*, *ID1*, *ID3,* and *MYC*), protooncogenes (*FOS*, *JUN*, *FOSB,* and *JUNB*), early growth response proteins (*EGR1* and *EGR3*) and a neural crest cell marker (*SNAI1*) (Fig. 3H). The high expression status of some of these factors in the AnLTs was also confirmed using IHC staining (Fig. 3I). The highly expressed transcriptional (*FOS*, *FOSB*, *JUN,* and *JUND*) and inflammatory (*CCL2* and *NFKBIA*) factors in SC2 were found to be enriched in both IL-17 and TNF inflammatory signaling pathways (Fig. 3D). Therefore, SC2 may provide the niche required for antler full regeneration as the stem cell-based processes.

The SC3 subcluster was roughly equally resident in both the AnLTs and FP (Fig. 3C). Interestingly, those highly-expressed genes were mainly involved in focal adhesion, regulation of actin cytoskeleton, ECM-receptor interaction, and hippo signaling pathway (Fig. 3D). Thus, SC3 may be related to the maintenance of basic metabolism and anatomical structure of the periosteum.

The SC4 subcluster was dominated in the AcAP with a negligible proportion in the FP (Fig. 3C). Importantly, the androgen hormone receptor (*AR*) was highly expressed in SC4, and one of its downstream genes (Yuan et al. 2010), the relaxin family peptide receptor 2 (*RXFP2*), specifically expressed in SC4 (Fig. 3E). It is known that antler generation is triggered by high levels of circulating androgens (Li et al. 2003). Thus, SC4 may be the subpopulation that is responsible for receiving androgen signals. In addition, both previous transcriptomic and proteomic studies showed that both Wnt signaling (Ba et al. 2019b; Mount et al. 2006) and PI3K-Akt signaling (Li et al. 2012; Liu et al. 2018) are activated during the phase of antler lineage cell activation (Fig. 3D). Overall, SC4, together with SC2 and SC3, may constitute the core cell types for antler full regeneration.

### Endothelial and mural cells each are heterogeneous at transcriptional level

**Fig. 4:**
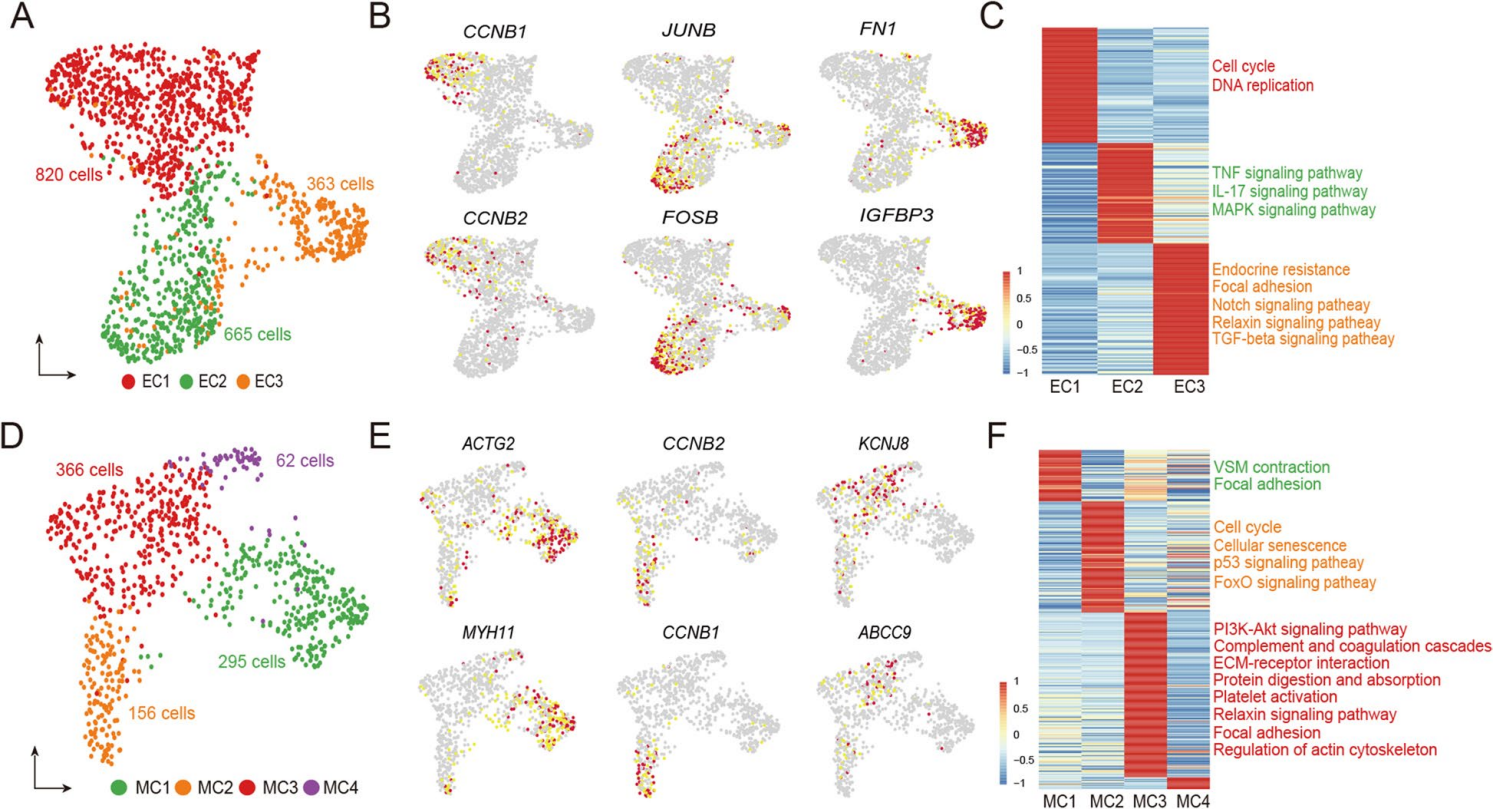
Cell subclusters in vascular endothelial and mural cells. **A** UMAP plot to visualize three endothelial subclusters (EC1, EC2, and EC3). **B** UMAP plots to show the highly expressed marker genes of three endothelial subclusters. **C** Heatmap plot to show the average expression levels of the DEGs in the three endothelial subclusters. The descriptions right to the heatmap are the top KEGG enriched pathways (adjust *p* value < 0.05). **D** UMAP plot to visualize the four mural subclusters (MC1, MC2, MC3, and MC4). **E** UMAP plots to show the highly expressed marker genes of four mural subclusters. **F** Heatmap plot to show the average expression levels of the DEGs in the four mural subclusters. The descriptions right to the heatmap are the top KEGG enriched pathways (adjust *p* value < 0.05)

Angiogenesis and vascular remodeling are key components of tissue regeneration. Rapidly full regeneration and growth of the antler also require support from a dense vascular network and two main cell types: endothelial cells and mural cells. Firstly, we reclustered endothelial cells to gain deeper insight into their cell characteristics and identified four subclusters (Fig. S4A). One of these subclusters (Fig. S4B) was lymphatic endothelial cells characterized by high expression of lymphatic marker genes, *MMRN1* and *FLT4* (He et al. 2018), and was excluded from the following analysis. Next, we further clustered the remaining cells into three subclusters (EC1, EC2, and EC3) (Fig. 4A and Table S6). The EC1 highly expressed cell cycling markers (*CCNB1* and *CCNB2*; Fig. 4B) and was identified to be the proliferative endothelial progenitor cells. The EC2 highly expressed *JUNB* and *FOSB* relating to IL-17 and TNF inflammatory signaling pathways (Fig. 4C and Table S7). Interestingly, these pathways were also involved in the SC2 in the *THY1*^+^ cells. These results suggest that the EC2 likely has functions similar to SC2 to provide the inflammatory niche required for vascular network development. The EC3 highly expressed Fibronectin 1 (*FN1*) and its binding protein, insulin-like growth factor binding protein 3 (*IGFBP3*) (Beattie et al. 2009). FN1 is a cytoskeletal protein and is mainly found in mesenchymal cells (Sudo et al. 2013). It is reported that FN1 promotes the opsonization of tissue debris, migration, proliferation, and contraction of cells and is involved in angiogenesis during tissue regeneration (Stoffels et al. 2013). The pathway enrichment analysis displayed that endocrine resistance, Notch, Relaxin, and TGF-beta signaling pathways were involved in the EC3 (Fig. 4C), suggesting that these developmental pathways may play important roles in endothelial cell expansion.

Mural cells consist of vascular smooth muscle cells (VSMC) and pericytes. We reclustered mural cells into four subclusters (MC1-MC4; Fig. 4D and Table S6): MC1, highly expressed markers (*MYH11* and *ACTG2*) of VSMC (Fig. 4E); MC2, highly expressed mitotically-active genes *CCNB1* and *CCNB2* and was defined to be the proliferative progenitor cells; MC3, highly expressed markers (*KCNJ8* and *ABCC9*) of pericytes (He et al. 2018). MC4 only contained 62 cells that were highly expressed 9 genes (Table S6), but these highly expressed genes did we not permit us to define the cell state/type. The pathway enrichment analysis further supported our classification of these mural cell subclusters (Fig. 4F and Table S7): for instance, MC3 was involved in PI3K-Akt signaling, focal adhesion, ECM-receptor interaction, protein digestion and absorption and regulation of actin cytoskeleton, which is consistent with a previous report that pericytes have the attributes of mesenchymal cells (Crisan et al. 2008).

To trace cell lineages during the differentiation of endothelial and mural progenitor cells, respectively, we performed pseudotime trajectory analysis to identify bifurcation points. For the trajectory of endothelial cell differentiation, proliferative progenitor cells of EC1 were located at the beginning of the trajectory path, whereas EC2 and EC3 were in the two terminal ends (Fig. S4C), suggesting these three subclusters were in different differentiation states along the trajectory. For the trajectory of mural cell differentiation, proliferative progenitor cells of MC2 were found at the starting point (Fig. S4D). It is reported that pericytes have the potential to transdifferentiate into VSMC (Crisan et al. 2008). As expected, we found that MC1 (VSMC) and MC3 (pericytes) were intermixed on the trajectory of these lineages, supporting this potential characteristic of pericyte transdifferentiation. In summary, these heterogeneous endothelial and mural cells may play a pivotal role in the formation of a vascular network during antler full regeneration.

### Trajectories of the *THY1*^+^ cell differentiation in antler generation and regeneration

**Fig. 5:**
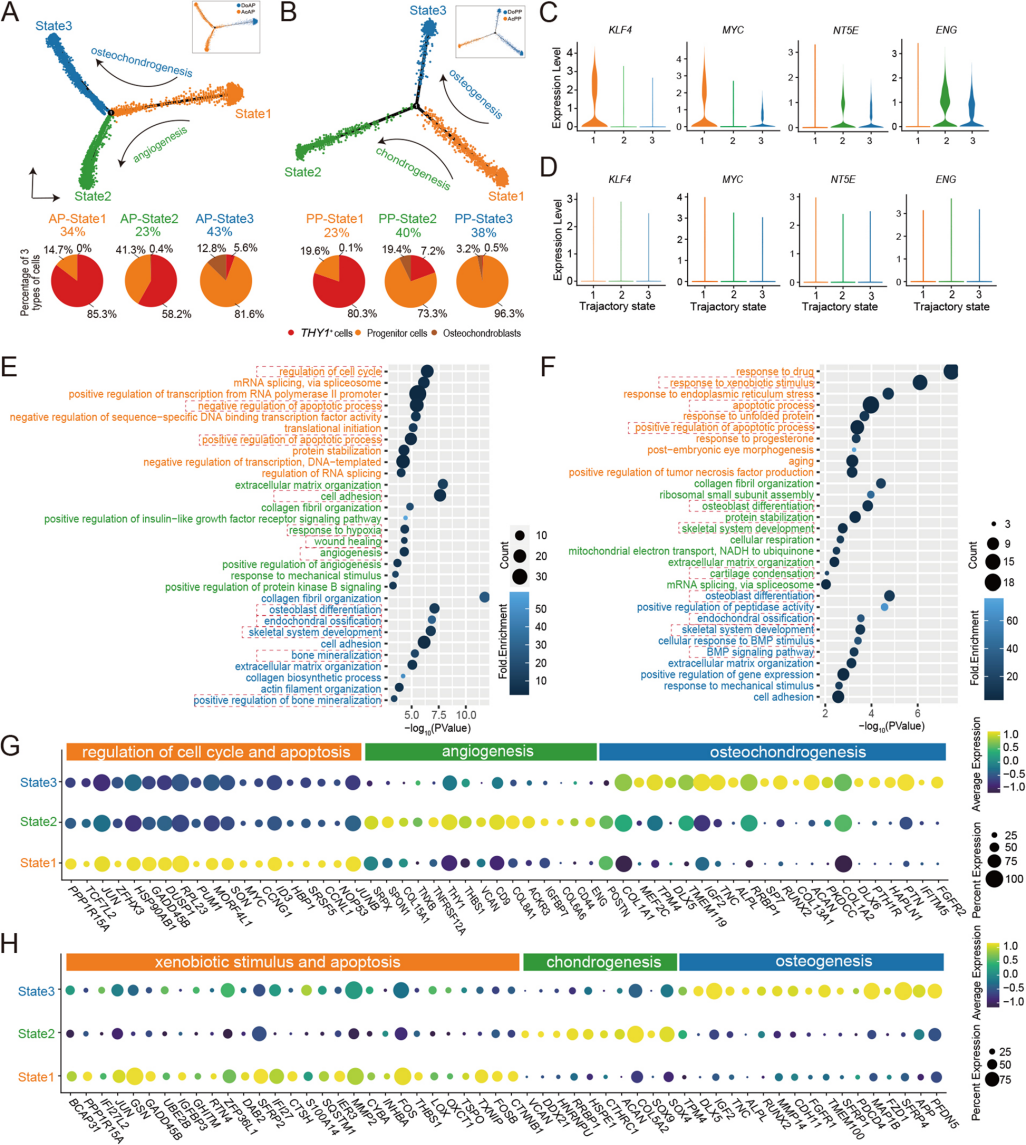
Pseudotime trajectories of *THY1*^*+*^ cell differentiation in antler generation and regeneration. States along pseudotime trajectory of *THY1*^*+*^ cell differentiation: **A** the trajectory of DoAP and AcAP; and **B** the trajectory of DoPP and AcPP. Pie charts to show the percentages of *THY1*^*+*^ cells, progenitor cells, and osteochondroblasts over the total number of cells at each state, respectively. Violin plot to show the expression levels of embryonic stem cell marker genes (*KLF4* and *MYC*) and mesenchymal marker genes (*NT5E* and *ENG*) at each state along **C** the trajectory of DoAP and AcAP and **D** the trajectory of DoPP and AcPP. Bubble plot to show the top terms of DAVID gene ontology (GOTERM_BP_DIRECT) at each state along **E** the trajectory of DoAP and AcAP and **F** the trajectory of DoPP and AcPP. The key terms are highlighted in dashed box. **G** Dot plot to show the expression levels of genes associated with regulation of cell cycle and apoptosis, angiogenesis, and osteochondrogenesis. **H** Dot plot to show the expression levels of genes associated with xenobiotic stimulus and apoptosis, chondrogenesis and osteogenesis

Antlers and pedicles are organs of cartilage/bone and directly formed from the proliferation and differentiation of the *THY1*^+^ cells. Therefore, we used pseudotime trajectory analysis in an unbiased manner to compare the initial differentiation processes for three cell types: *THY1*^+^ cells, progenitor cells, and osteochondroblasts during antler generation (Fig. S5A) and regeneration (Fig. S5B). Three states were identified for each differentiation trajectory: AP-state 1, AP-state 2, and AP-state 3 for antler generation (Fig. 5A); and PP-state 1, PP-state 2, and PP-state 3 for antler regeneration (Fig. 5B). For antler generation, we compared percentage of each cell type at each state, and AP-state 1 was found at the starting point along trajectories based on the percentage value of the *THY1*^+^ cells. Compared to AcAP, DoAP had a more abundant AP-state 1. Two highly expressed pluripotent stem cell markers (*KLF4* and *MYC*) were found in AP-state 1 (Fig. 5C), which was consistent with their high expression in the SC2 dominated in the DoAP (Fig. 3E). Two mesenchymal cell markers (*NT5E* and *ENG*) were found to be upregulated at AP-state 2 and AP-state 3 (Fig. 5C), resembling the signature of mesenchymal cells, and especially, *NT5E* and *ENG* were more highly expressed at AP-state 2 relative to AP-state 3. Interestingly, the *KLF4, MYC, NT5E,* and *ENG* were not found to be expressed during antler regeneration (Fig. 5D), suggesting that although antler generation and regeneration both are derived from differentiation of the *THY1*^+^ cells, the degree of stemness of the initial stem cells for these two processes are different, and the AP-*THY1*^+^ cells for antler generation were more primitive and resembling those that reside in the niche of embryonic tissue.

Upregulated genes at AP-state 1 were involved in the regulation of the cell cycle and apoptosis (*CCNG1*, *ID3, MYC,* and *CCNL1,* Figs. 5E, G, and Table S8). The high proportions of progenitor cells and osteochondroblasts at AP-state 2 and AP-state 3, in comparison to AP-state 1, indicate that cells at these states have differentiated toward mesenchymal lineage along the *THY1*^+^ cell differentiation trajectory. The upregulated genes at AP-state 2 were found to participate in cell adhesion, response to hypoxia, wound healing and angiogenesis (*THY1*, *THBS1*, *CD9*, *IGFBP7*, *CD44,* and *ENG*; Figs. 5E, G and Table S8). AP-state 3 was annotated to participate in osteoblast differentiation and endochondral ossification (*POSTN*, *IGF2*, *ALPL*, *RUNX2*, *SP7, PTH1R,* and *ACAN*; Figs. 5E, G and Table S8). Like AP-state 1, upregulated genes at PP-state 1 were involved in the regulation of apoptosis (*MMP2* and *CTSH*; Figs. 5F, H and Table S8). Analysis of the *THY1*^+^ cell differentiation trajectory in antler regeneration also found ramifications of two states (PP-state 2 and PP-state 3): PP-state 2 differentiated toward chondrogenesis (*VCAN*, *ACAN,* and *SOX9*) and PP-state 3 toward osteogenesis (*ALPL*, *RUNX2,* and *DLX5*; Figs. 5F, H and Table S8). PP-state 2 dominated in the AcPP, and PP-state 3 dominated in the DoPP. The results of the trajectory further showed that differentiations of the *THY1*^+^ cells are different during antler generation and regeneration.

Our IHC staining results further confirmed that the AcAP tissue highly expressed POSTN (Periostin), IGF2, and THBS1 proteins (Fig. S5C). POSTN encodes secreted extracellular matrix protein that functions in tissue development and regeneration, including wound healing, and this protein is reported to be required for bone repair and involved in the regulation of the skeletal stem cell niche within the periosteum (Duchamp de Lageneste et al. 2018; Rios et al. 2005). *IGF2*, a major fetal growth factor in mammals, is known to be involved in the regulation of fetoplacental development (DeChiara et al. 1990). In this respect, AP tissue has been considered as a postnatally-retained embryonic tissue (Li and Suttie 2001), and thus it might be expected that *IGF2* would play an important role in the regulation of the AnLT cell activation to form pedicles and antlers. Thrombospondin-1 encoded by THBS1 is reported to promote angiogenesis through interactions with a number of integrin heterodimers in vascular cells (Chandrasekaran et al. 2000). Indeed, some integrin-encoding genes (*ITGA5*, *ITGA6*, *ITGA7*, *ITGB1,* and *ITGB4*) were found to be highly expressed in endothelial cells and/or mural cells (Fig. S5D). Therefore, THBS1 is likely to serve as a pro-angiogenic factor for the formation of a vascular network during antler full regeneration.

### AR/RXFP2 signaling is found to be firstly activated in the initiation of antler full regeneration

**Fig. 6:**
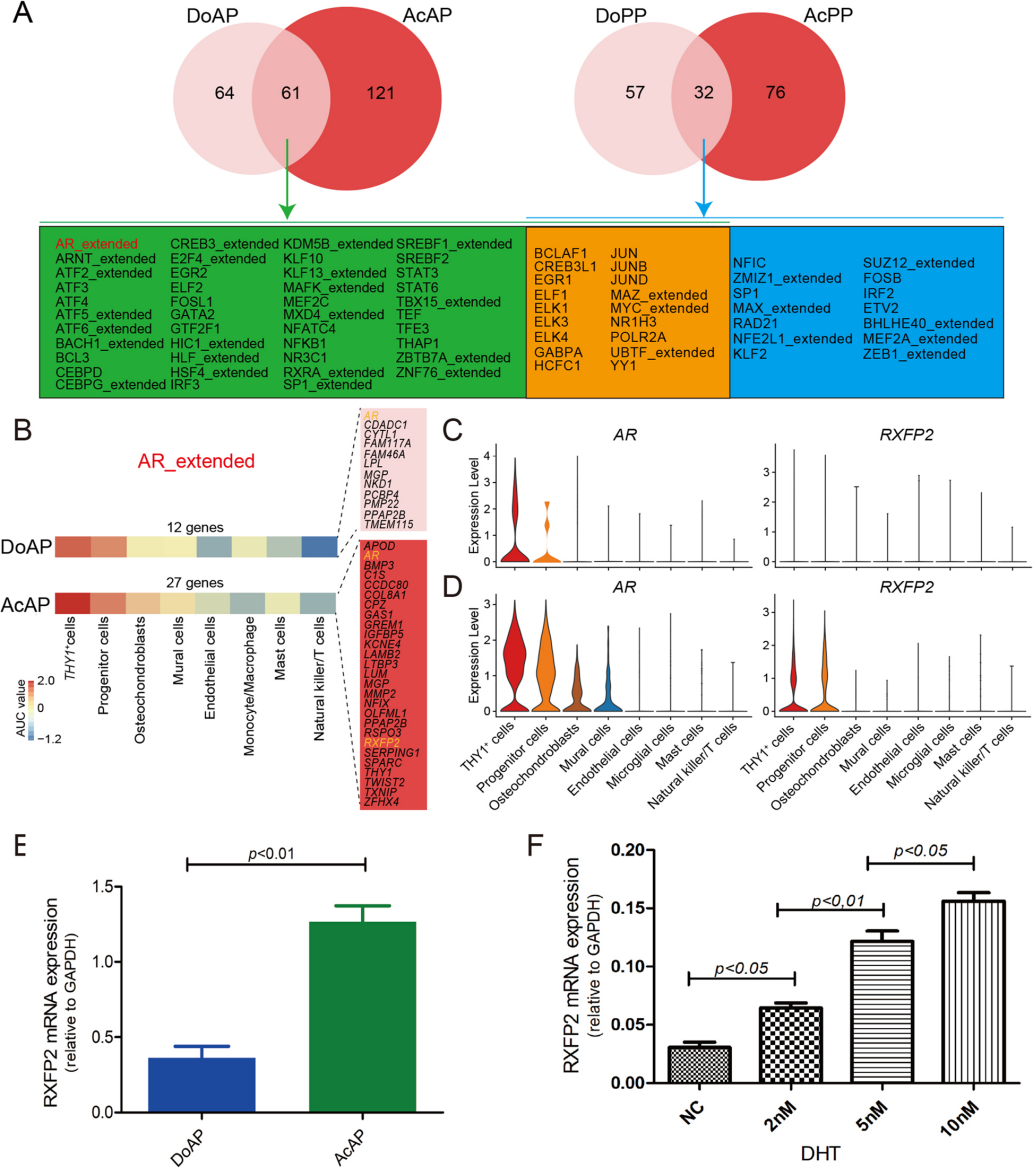
Regulons activated during the initiation of antler generation and regeneration. **A** Venn diagram to show the activated regulons during differentiation from DoAP to AcAP, and those from DoPP to AcPP. The 61 shared regulons of the DoAP and AcAP (green and orange background), and the 32 shared regulons of the DoPP and AcPP (blue and orange background), and the 18 shared regulons of antler generation, and regeneration are in orange background. **B** Heatmap plot to show the activity of AR regulon in the eight-cell types of the DoAP (upper panel) and AcAP (lower panel). The activity of AR regulon was calculated using the area under the curve (AUC). Note that activity of AR regulon was significantly higher in *THY1*^+^ cells and progenitor cells. AR regulon in the AcAP includes 27 genes (red background) and in the DoAP includes 12 genes (pink background). *RXFP2* and *AR* are in orange. Violin plots to show the expression levels of *AR* and *RXFP2* in AR regulon in the DoAP (**C**) and in the AcAP (**D**). **E** Expression levels of *RXFP2* mRNA in the AcAP and DoAP using qRT-PCR. Value: mean ± S.E.M. *n*=3. **F** Influence of DHT on the expression levels of RXFP2 mRNA in the AP cells based on qRT-PCR analysis. Different concentrations (2, 5, 10 nM) of DHT were applied for 24 h. Value: mean ± S.E.M. *n*=3, NC: Normal control

TO further characterize the regulatory network at the transcriptional level of each cell type for antler generation and regeneration, we analyzed their regulons (co-expressed transcription factors and their putative targets) and identified eight cell-type specific regulons in the AnLTs (Figs. S6, S7A). The results showed that more regulons were detected in the activated tissues (AcAP: 182; AcPP: 133) relative to dormant tissues (DoAP: 125; DoPP: 89; Fig. 6A). We also found that 61 co-activated regulons in antler generation were more than those (32) in antler regeneration. Notably, of these co-activated regulons, AR regulon was found to be the activated signal only for antler generation (Fig. 6A), and furthermore, AR regulon included 27 target genes in the AcAP but only 12 target genes in the DoAP (Figs. 6B; S7B, C, D). More expressed genes in the AR regulons in the AcAP than in the DoPP suggest target genes in the AR regulon are AP activation-dependent. The previous findings *in vivo* studies indicate that androgen hormones are the signal triggering the initiation of pedicle/antler generation (Suttie et al. 1995); *in vitro* studies showed that AR was clearly localized in the AP cells (Li et al. 1994; Li et al. 2001). Conversely, AR regulon was not detected in the tissues that participate in antler regeneration (DoPP and AcPP), which is also consistent with the *in vivo* findings that antler regeneration can only be activated when the circulating androgen hormones are decreased to an almost undetectable level (Li et al. 2003; Suttie et al. 1995). We further found that the *THY1*^+^ cells and progenitor cells in the AcAP highly expressed *AR* and *RXFP2* than those in the DoAP (Figs. 6C, D). Results of qRT-PCR also confirmed that *RXFP2* mRNA level significantly increased in the AcAP compared with that in the DoAP tissues (Fig. 6E). It was reported that *RXFP2* is a downstream target gene of *AR* (Yuan et al. 2010). To further confirm that *RXFP2* mRNA expression is causally related to the change in androgen hormone level in the AP cells, we carried out an *in vitro* assay. The results showed that the mRNA expression level of *RXFP2* in the AP cells increased in a dose-dependent fashion with the increase in DHT concentration (Fig. 6F).

## Discussion

IT is well established that blastema formation results from the dedifferentiation of the differentiated cell types on the amputation plane back to embryonic-like cells (Mescher 1996), but these cell types retain the memory of their origin when they re-differentiate (Kragl et al. 2009). Therefore, a blastema consists of multiple different cell types, which has been confirmed by recent studies using scRNA-seq (Gerber et al. 2018; Leigh et al. 2018; Li et al. 2021). Deer antlers are the only case of mammalian epimorphic full regeneration, which is realized through the differentiation of pluripotent/mesenchymal stem cells resident in the AnTLs (Li et al. 2007). To the best of our knowledge, this is the first study to assess the cellular composition in four types of the AnLTs, namely the DoAP, AcAP, DoPP, and AcPP, via scRNA-seq. Combined with previous histological and morphological data, our results demonstrate that antler full regeneration is consistent with a conceptual stem-cell-based regenerative process, which provides a useful genetic resource for mammalian organ regeneration.

We identified three core subpopulations of the *THY*^*+*^ cells (SC2, SC3 and SC4). The SC4 dominated in the AcAP, and highly expressed *AR*. It is known that pedicle development is triggered by high levels of circulating androgens (Li et al. 2003) and *AR* is a type of ligand-dependent receptor (Hu et al. 2002). Another gene, *RXFP2*, was also found to be exclusively expressed in SC4. Importantly, the previous study indicated that *RXFP2* is a downstream target gene of *AR* (Yuan et al. 2010). In the present study, AR/RXFP2 signaling is also found to be firstly activated in the initiation of antler generation. RXFP2 is found the sole factor that controls phenotype of sheep horns (Johnston et al. 2011) and thus RXFP2 may also be the indispensable factor that specifies phenotype of deer antlers, another type of ungulate headpieces. Thus, SC4 may be the subpopulation that is responsible for receiving androgen signals. Further study is needed to find out the underlying molecular mechanism of androgens regulating *RXFP2* and the exact role of *RXFP2* in antler full regeneration.

Androgens are known to effectively promote inflammation in wound healing (Gilliver et al. 2003), and trigger the initiation of pedicle (male secondary sexual characters) growth (Li et al. 2003). Therefore, potent androgens, together with the factors enriched in the two activated IL-17 and TNF inflammation pathways in SC2, may constitute the necessary milieu for pedicle to initiate and grow. These two inflammatory signaling pathways were also detected during regenerative wound healing over the digit tip stumps (Kisch et al. 2015), the rare example of regeneration in another mammalian genera. Therefore, SC2 may not only directly participate, but also provide the niche required for pedicle and antler formation as the stem cell-based processes. Indeed, the subcutaneous injection of a small amount of CCL2 (0.5 ml/region), a type of chemokine for macrophages (highly expressed in the SC2), to the presumptive pedicle growth region of a female sika deer (naturally antler-less although it possesses an AP, but lacks a source of potent androgen stimulation) can successfully stimulate the female deer to grow pedicles and antlers (Wang et al, unpublished).

To our surprise, SC1, the only FP specific subpopulation, highly expressed the gene *AKR1C4.* This gene encodes an enzyme that catalyzes the conversion of more potent forms of androgen (e.g., dihydrotestosterone) to the less active forms (e.g. 5-alpha-androstan-3-alpha) (Penning et al. 2000; Steckelbroeck et al. 2004). Consequently, SC1 may mediate a process of attenuating the potency of androgen hormone signals to ensure that the non-antlerogenic tissues (FP) are unable to respond to the type and level of androgens that would otherwise stimulate the DoAP to initiate pedicle formation. In the case of DoPP, besides the low level of circulating androgen hormones in the milieu, the intermediate level of AKR1C4 activity may also be required to provide a necessary niche for antler regeneration to take place. In the case of PP for antler regeneration to take place, besides the low level of circulating androgen hormones in the milieu, the intermediate level of AKR1C4 activity may also be required to provide a necessary niche. Our present results are consistent with the *in vivo* findings that DoAP is activated by strong androgen stimulation to form the pedicle via the AcAP (Li et al. 2003) and that the DoPP can only begin to regenerate the antler when the androgens have declined to an almost undetectable level (Suttie et al. 1995).

Proliferation cell type composition in a tissue undergoing rapid growth is currently not fully understood. Deer antlers have been claimed as the fastest growing animal tissue and their growth rate can reach 2 cm/day (Goss 1983). It is the rapid proliferation of the inner sublayer of AcPP cells that drives antler regeneration with the fast-forming blood vessels commensurate the pace of this growth rate. In our earlier work, we were not able to define the exact types of the mitotically-active cells. In the present study using the scRNA-seq approach, we identified clearly that the cells in the inner sublayer of the AcPP were progenitor cells (that have lost critical stem cell attributes but had not yet started to differentiate). Consequently, the intensive proliferation of the progenitor cells and blood vessel cells constitute the main cell source for building up the tissue mass that adds to the growing antler tip through appositional growth. Identification of the potent growth factors or unique regulation systems that can stimulate antler progenitor cell proliferation to that speed would have potential clinical application.

It appears that apoptosis may function as one of the intrinsic factors triggering initial tissue regeneration, as it is known that apoptosis-induced compensatory proliferation plays an essential role in the tissue homeostasis of multiple organisms (Brock et al. 2019; Colitti et al. 2005; Jiang et al. 2009). Our results showed that upregulated genes at AP-state 1 and PP-state 1 were involved in the regulation of the cell cycle and apoptosis, which probably reflects the phenomenal rate of morphogenesis and tissue remodeling that takes place in antler full regeneration.

Angiogenesis and initiation of regeneration are two highly coupled processes (Liu et al. 2021). Initiation of pedicle growth starts from the proliferation of the AP cells, and the proliferating AP cells differentiate into the mixture of osteoblasts and chondroblasts to build up initial pedicle tissue (Li and Suttie 1994a); at the same time, an ample blood vessel network is rapidly formed around the early growing pedicle tissue, that is why transplanted AP tissue can readily survive and form ectopic or xenogeneic antlers, but PP tissue cannot do so due to the insufficient blood supply (Li et al. 2009a; Li et al. 2010; Wang et al. 2022). At the DoPP state, PP cells slowly proliferate and differentiate into osteoblasts and then osteocytes to thicken the pedicle bone in an appositional way. In contrast, at the AcPP state, activated PP cells almost all differentiate into cartilage-lineage cells and build up entire regenerating antler blastema within days (Kierdorf et al. 2003; Li et al. 2004, 2005).

Our results also raise a number of fundamental questions, including how a heterogeneous cell population resident in the niche of periosteum/perichondrium communicates and works in concert to 1) initiate an organ formation (antler generation) in the postnatal life of an animal (puberty) and 2) fully and repeatedly regenerate an entire complex mammalian organ (antler regeneration)? 3) response to androgen hormones for the proliferation and control phenotype by linking to *RXFP2* gene. Answers to these questions would undoubtedly open up new avenues to reveal the molecular mechanism underlying deer antler development specifically, but more importantly, to understand how nature has solved the problem of mammalian organ generation/regeneration in the field of regenerative medicine in general.

## Methods

### Collection of deer tissue samples

The DoAP was sampled from three 7-month-old deer (prepubertal; before pedicles start to grow), and the AcAP and FP from three 10-month-old deer (pubertal; pedicle growth has initiated; and FP was sampled at this stage, as we believe FP should still be comparatively quieter to the activated AP); the DoPP and AcPP were from three 2-year-old deer (first round of antler regeneration from the fully-grown pedicles). Methods for the collection of these deer tissues have been reported in detail elsewhere (Li et al. 2003). Briefly, for DoAP and AcAP, the deer were slaughtered in a commercial abattoir, and each head was brought into the cell culture laboratory. The presumptive pedicle growth region was located using the frontal crest as a landmark. The skin covering the crest was surgically removed to expose the periosteum (DoAP or AcAP), an oval-shaped incision was made surrounding the crest using a scalpel and the periosteum was peeled off from the crest using rat-tooth forceps (Fig. S1A). For the FP, the skin covering the forehead and facial regions was opened to expose the FP, which was then peeled off using rat-toothed forceps. For DoPP and AcPP, appropriate growth stages of pedicles were surgically removed from their base immediately after the hard antler buttons naturally dropped off, and brought into our laboratory. For the DoPP, the enveloping skin was removed through the longitudinally cut incisions on the pedicle shaft to expose the periosteum, which was then removed by cutting longitudinal strips (around 0.5 cm wide) using a scalpel and peeled off using forceps (Fig. S1C); For the AcPP, newly formed velvet skin covering the early growing antler bud (blastema) removed to expose the underlying tissue, the outmost layer (equivalent to the reserve mesenchyme layer in an antler growth center) removed (Fig. S1F).

### Preparation of single-cell suspension

Each tissue sample was transferred to a sterile 10-cm culture dish and cut into < 1 mm^3^ segments before being transferred to a 50-ml centrifuge tube. Each tissue type was then digested in the DMEM (Sigma-Aldrich, USA) containing 100 μg/ml collagenase type I and 100 μg/ml type II (Invitrogen, USA) for 40-80 min at 37 °C with intermittent shaking. When more than 20,000 cells were released, digestion was stopped by adding 10% (V/V) fetal bovine serum (Gibco, USA). Digests were passed through a 70-μm filter, and cells were collected by centrifugation (500g for 5 min at 4 °C). To remove red blood cells, we treated cell pellets with 1 × red blood cell lysis buffer (Beyotime, China) for 5 min at room temperature and washed with PBS. Live cell numbers were counted again using AO/PI double fluorescence staining kit (Beyotime, China).

### Single-cell library construction and sequencing

Single-cell library construction and RNA sequencing were performed at Capitalbio Technology Corporation (Beijing, China) using the 10X Genomics system (Pleasanton, CA). Briefly, 2,000 to ~10,000 single cells were obtained from the five tissue types (mixed three samples/each tissue type). For each experiment, cells were diluted following the manufacturer’s recommendations and mixed with totally mixed buffer before being loaded into 10X Chromium Controller using Chromium Single Cell 3’ Reagent v2 reagents. Each sequencing library was prepared following the manufacturer’s instructions, with 13 cycles used for cDNA amplification. Then ~100 ng of cDNA from each library was used for amplification through 12 cycles. The resultant libraries were sequenced on an Illumina HiSeq 4000.

### Single-cell data preprocessing

Cell Ranger (version 3.0.2, 10X Genomics) was used to perform alignment and read counting with default parameters by mapping onto the sika deer genome (CNCB: GWHANOY00000000). The Unique Molecular Identifiers (UMI) count matrix generated by Cell Ranger was further processed by Seurat package v3.2.1 (Butler et al. 2018). We set three criteria to gain high-quality cells: 1) only cells that expressed more than 200 genes were included, and only genes expressed in at least 3 single cells were included for further analysis; 2) cells with extreme abnormality in gene number were excluded (the upper limit of filtering is Q3 + 1.5 * IQR, and the lower limit is Q1 - 1.5 * IQR); and 3) cells with a mitochondrial gene percentage over 10% discarded. The different samples were integrated using “IntegrateData” function in Seurat. In total, 13,267 genes across 28,213 single cells were gained for subsequent analysis.

### Dimension reduction and clustering

Principal component analysis (PCA) was performed on the integrated data above, and the first 30 PCs were chosen for dimension reduction and clustering. In this process, "RunUMAP" function in Seurat was used to reduce the dimension, and cell clustering was achieved by "FindNeighbors" and "FindClusters" (resolution = 1.2) functions, respectively.

### Cell type annotation and differentially expressed genes analysis

Due to there being only a few studies on deer cell types, we used markers of human and mouse cells to define cell types. Differentially expressed genes (DEGs) of different cell types were calculated by “FindAllMarkers” function in Seurat, and the thresholds for DEGs screening were: *p*-value < 0.01 and log_2_FoldChange > 0.5 (cell types) or 0.25 (cell subpopulation). In the *THY1*^+^ cell type, cluster 6 was excluded from subsequent analysis due to low transcript (UMI) counts.

### RNA preparation, bulk RNA-seq library construction and sequencing

Around 0.5 g fresh tissues from each sample (three individuals, each tissue type) were immediately frozen in liquid nitrogen and then stored at -80 °C for RNA extraction. These tissues were rapidly ground into a fine powder using a Freezer/Mill 6770 (SPEX CertiPrep Ltd., USA). Total RNA was extracted from each sample powder using a Trizol reagent (Invitrogen Inc., USA) according to the manufacturer’s procedure. RNA quality was confirmed using Bioanalyzer with a minimum RNA integrity number of 7.0. A total of 1.0 µg RNA from each sample was used as input for the Illumina TruSeq RNA Library Preparation Kit v3, and libraries were constructed according to the manufacturer’s instructions. The libraries were sequenced on an Illumina HiSeq 4000.

### DEGs of bulk RNA-seq data

Quality control and preprocessing of raw reads were performed using Fastp v0.11.8 (Chen et al. 2018). The clean reads mapping and gene expression level quantification were performed using the workflows of HISAT2, StringTie, and DESeq2 (Pertea et al. 2016). Briefly, reads were aligned against the sika deer genome (CNCB: GWHANOY00000000) using HISAT v2.1.0 (Kim et al. 2015). Gene expression abundance in each sample was estimated using StringTie v 2.0 (Pertea et al. 2015). The Python script of prepDE.py was used to extract the read count information of each gene from the coverage values estimated by StringTie. After the results of prepDE.py returned, DEGs between two groups (Quiescent vs. Activated) were detected using DESeq2 v1.18.1 (Love et al. 2014) based on |log_2_FoldChange| ≥ 1.5 and Benjamini Hochberg *p* value < 0.01.

### Correlation analysis

The similarities/differences between the five tissue types were investigated using correlation analysis based on RNA-seq and scRNA-seq data separately. For the RNA-seq data, the Pearson correlation was calculated based on the normalized expression matrix. As for the scRNA-seq data, we first obtained the pseudo-bulk matrix through the average expression of each sample and then applied the Pearson correlation analysis as the RNA-seq data.

### Cell cycle phase classification

We applied the “cyclone” function in scran v1.14.6 (Lun et al. 2016) to divide cells into cell cycle phases based on the pair-based prediction method. Pairs of markers were assessed using the classification procedure. In brief, the proportion of all marker pairs was calculated to assess whether the expression of the first was greater than the second in the gene pairs, and the proportion was then converted to a score. Cells with a G0/1 or G2/M score higher than 0.5 were assigned to the G0/1 or G2/M phase, respectively (if both were higher than 0.5, the higher score was used for the assignment). Cells were assigned to S phase when both G0/1 and G2/M scores were below 0.5.

### Gene set enrichment analysis

For the generated DEGs of bulk RNA-seq data, gene set enrichment analysis (GSEA) was performed using GSEA tool v4.1.0 (Bean et al. 2020) based on the Molecular Signatures Database C5 v7.1 with a 1,000 per mutation number. We set the cut-off criteria as gene size ≥ 15 and nominal *p*-value < 0.005. Additionally, DAVID v2022q2 (Sherman et al. 2022) was used to obtain significant terms of gene ontology (GOTERM_BP_DIRECT) with an adjusted Fisher exact *p*-value < 0.05. KEGG enrichment analyses were carried out using clusterProfiler v3.14.3 (Yu et al. 2012), and the results of adjusted *p*-value < 0.05 were considered significant. KEGG pathways that are disease-related were not considered.

### Pseudotime trajectory analysis

Monocle2 v2.14.0 (Trapnell et al. 2014) was used to perform pseudotime trajectory analysis on three types of cells (*THY1*+ cells, progenitor cells, and osteochondroblasts). The UMI counts matrix was used as input. Furthermore, to use as little prior knowledge to study the differentiation process, we performed this analysis in a completely unsupervised way. In detail, the function “dispersionTable” was used to identify the high dispersion genes, and the input ordering genes were selected by the following threshold: mean_expression ≥ 0.5 & dispersion_empirical ≥ 1 * dispersion_fit. Then each cell was ordered by “orderCells” function and assigned a “pseudotime” value, and genes that change as a function of pseudotime were calculated using “differentialGeneTest” function. The gene change trend was visualized by “plot_genes_in_pseudotime”.

### Regulon activity analysis

The transcriptional factor regulation network was predicted by SCENIC v1.1.3 (Aibar et al. 2017). First, the co-expression module was deduced. Second, Rcis Target database was used to construct the gene regulation module (regulon). Third, the area under the curve (AUC) was calculated to indicate the network activity of each cell.

### Enzyme-linked immunosorbent assay

The activity of AKR1C4 was measured using the AKR1C4 enzyme-linked immunosorbent assay (ELISA) Kit (MlBio, China) according to the manufacturer’s procedure. Briefly, culture supernatant was collected from the cells 48 h after seeding and centrifuged for 20 min at room temperature (5,000 g). The supernatant was added to the wells of the enzyme-labeled coating plate (50 μl each well), with triplicates for each sample. Then 100 μl of enzyme-labeled reagent was added to each well. The plate was sealed with a sealing film and incubated at 37 °C for 60 mins. The plate was washed with PBS 5 times, and 50 μl of developer A and B was added to each well, respectively. The plate was shaken gently to mix and incubated at 37 °C for 15 mins in the dark. Finally, 50 μl stop solution was added to each well, and the absorbance (OD value) at 450 nm was measured with a microplate reader (Thermo, USA).

### Western blotting

Total proteins were extracted using Radio Immunoprecipitation Assay lysis buffer (Beyotime, China). Proteins (20µg/lane) from each tissue sample were separated by 12% sodium dodecyl sulphate-polyacrylamide gel electrophoresis (SDS-PAGE) and transferred to polyvinylidene fluoride membranes. Membranes were blocked with 5% (w/v) skimmed milk powder and immunoblotted with suitably diluted primary antibody AKR1C4 (Cat No: A7430, ABclonal, China) at a dilution of 1:200 followed by secondary antibodies (goat anti-rabbit IgG; Cat No: SE134, Solarbio, China) at dilution of 1:500 conjugated with horse radish peroxidase. Bands were visualized using enhanced chemiluminescence detection reagents (Thermo, USA) applied to Chemiluminescent Imaging System (Tanon, China). The quantification of western blot bands was carried out using ImageJ software v2.1 and normalized to GAPDH.

### Immunohistochemistry

Paraffin-embedded deer tissue sections (2-3 mm in thickness) were de-paraffinized and rehydrated. Endogenous peroxidase was quenched with 3% H_2_O_2_ for 5 min. Antigen retrieval was performed by boiling in 10 mM sodium citrate buffer (pH 6.0) for 10 min. The non-specific binding sites were blocked in PBS plus 10% normal goat serum for 30 min and then incubated with primary antibodies at dilution of 1:50, including KLF4 (Order No: bs-1064R, Bioss, China), JUN (A11378, ABclonal, China), MYC (ENT0990, Elabscience, China), SNAI1 (A11794, ABclonal), JUND (A11955, ABclonal), FOS (A0236, ABclonal), PCNA (A0264, ABclonal), THBS1 (A2125, ABclonal), POSTN (A14556, ABclonal) and IGF2 (A2086, ABclonal) for 2 h at 37 ℃. For isotype control, the primary antibody was replaced by rabbit/mouse IgG (ab172730 and ab37355, Abcam, UK) at dilution of 1:100. After rinsing in PBS, sections were incubated with secondary antibody conjugated with HRP (AS014 and AS003, ABclonal for 30 min. After rinsing in PBS, all sections were stained with DAB chromogen reaction solution (Maxim, Fuzhou, China). The sections were then counterstained with haematoxylin and scanned with PreciPoint M8 (Meyer, Germany).

### Testosterone treatment

Released AP cells were cultured in a medium (DMEM + 10% FBS) in 24-well plates (2.5×10^5^ cells/well) at 37 °C with 5% CO_2_, according to our previous study (Wang et al. 2019). AP cells were treated with 2, 5, and 10 nM 5alpha-dihydrotestosterone (5α-DHT; Sigma, Germany) for 24 h. The cells were collected to measure the expression levels of *RXFP2* using qRT-PCR.

### cDNA synthesis and quantitative real-time PCR (qRT-PCR)

The standard RNA was used to reverse transcribe into cDNA by using PrimeScript^TM^ RT reagent Kit (TaKaRa, China). The primers used for amplifying *RXFP2* cDNA were 5’-GCTGAAAACACGACTCACGC-3’ (forward) and 5’-ACTGCAAGCTCTCCATCCAC-3 (reverse). The qRT-PCR analysis was performed as described previously (Ba et al. 2019b).

### Statistical analysis

Analysis of data was done using GraphPad software v5.0. Data were presented as mean ± standard error of mean (S.E.M.). Significant differences (at least *p*-value < 0.05) between the two groups were determined using the two-tailed independent samples t-test. Significant differences among three or more groups were determined using a one-way analysis of variance, followed by Tukey’s post hoc test.

## Supplementary Information

**Additional file 1: FigureS1**. Antler tissue types and their roles in antler development. (A) Antlerogenic periosteum (AP; arrow).(B) Ectopic antler (arrowhead) formed from the transplanted AP, whereas the presumptive region failed to develop pedicle and antler after missing the AP (arrow); (C) pedicle periosteum (PP; arrow). (D) PP-less pedicle failed to regenerate an antler (arrow), although the contralateral side intact pedicle gave rise to a 3-branched-antler. (E) A fully formed antler blastema (blackline: marked for the longitudinal cutting). (F) Longitudinally cut surface of the antler blastema (AcPP, activated PP tissue; GC, blastema growth center).

**Additional file 2: Figure S2.** (A) Pearson correlation coefficients between different tissue samples based on bulk RNA-seq (triplicates of each tissue type). (B) UMAP plot to show the cell clusters that were labeled by both color and number. (C) Heatmap plot to show the top DEGs of each cell cluster. (D) UMAP plots to show the expression patterns of partial marker genes in each cell type (also refer to Figure 1D). (E) UMAP plots to visualize eight cell types in eachof the five tissue types; the color regime for each cell type is the same to Figure1C.

**Additional file 3: Figure S3.** (A) UMAP plots to visualize the expression patterns of G2-phase specific marker genes, *CDK1 *and *CCNB1*. (B) UMAP plots to visualize the expression patterns of marker genes (*KLF4* and *MYC*) in the cells of each of five tissue types. (C) Pearson correlation coefficients between different *THY1*^+^cell subclusters. (D) UMAP plot to visualize the expression pattern of immune-related marker gene (*CD38*) in *THY1*^+^cell subclusters.

**Additional file 4: Figure S4.** (A) UMAP plot to visualize the distribution of endothelial cells in five tissue types (left) and in four cell subclusters (right). (B) UMAP plots to visualize the expression pattern of lymphatic marker genes (*MMRN1*and *FLT4*). (C) Pseudotime trajectory of three subclusters of endothelial cells. (D) Pseudotime trajectory of four subclustersof mural cells.

**Additional file 5: Figure S5.** Pseudotime trajectories of *THY1*^+^ cell differentiation in the DoAP and AcAP (A) and that in the DoPP and AcPP (B). (C) IHC staining of THBS1, POSTN and IGF2 in the AcAP. Note that these three factors were found to be highly expressed in the *THY1*^+^cells/progenitor cells. Positive staining of POSTN was mainly detected in the extracellular matrix. F, fibrous layer; C, cellular layer. (D) UMAP plots to show the expression pattern of genes, *ITGA5*, *ITGA6*, *ITGA7*, *ITGB1, *and *ITGB4*.

**Additional file 6: Figure S6.** Heatmap plots to show the regulon activities of the five tissue types.

**Additional file 7: Figure S7.** (A) UMAP plot to visualize the cell composition in the AcAP based on the activity of identified regulons. (B) Activation and expression profile of AR regulon in the AcAP (upper panel) and the DoAP (lower panel). Note that AR regulon in the AcAP (27 genes) was more active than that in the DoAP (12 genes). Heatmap plot to show the average expression levels of genes in AR regulon in the DoAP (C) and AcAP (D). *RXFP2* and *AR* are in red.

**Additional file 8: Table S1.** Quality control filtering of single-cell data of the five tissue types.

**Additional file 9: Table S2.** DEGs of the eight cell types (log_2_FoldChange > 0.5).

**Additional file 10: Table S3.** DEGs of activated vs. quiescent tissues from RNA-seq data.

**Additional file 11: Table S4.** DEGs among the five subclusters of *THY1*^+^ cells (log_2_FoldChange > 0.25).

**Additional file 12: Table S5.** KEGG enrichment analysis results of DEGs in each of four subclusters of *THY1*^+^ cells.

**Additional file 13: Table S6.** DEGs among subclusters in the vascular endothelial and mural cells (log_2_FoldChange > 0.25).

**Additional file 14: Table S7.** KEGG enrichment analysis results of DEGs in each of the subclusters in the vascular endothelial and mural cells.

**Additional file 15: Table S8.** DAVID gene ontology enrichment analysis results of DEGs at each state during antler generation andregeneration.

## Data Availability

The datasets generated during the current study are available in the CNSA and SRA under the accession number of CNP0002029 and PRJNA750429, respectively.

## Abbreviations

AP: Antlerogenic periosteum
PP: Pedicle periosteum
FP: Facial periosteum
scRNA-seq: Single-cell RNA sequencing
AnLTs: Antler lineage tissues
DoAP: Dormant antlerogenic periosteum
AcAP: Activated antlerogenic periosteum
DoPP: Dormant pedicle periosteum
AcPP: Activated pedicle periosteum
IHC: Immunohistochemistry
AKR1C4: Aldo-Keto Reductase Family 1 Member C4
AR: Androgen hormone receptor
RXFP2: Relaxin family peptide receptor 2
VSMC: Vascular smooth muscle cell
DMEM: Dulbecco’s Modified Eagle Medium
UMI: Unique Molecular Identifiers
UMAP: Uniform Manifold Approximation and Projection
DEGs: Differentially expressed genes
KEGG: Kyoto Encyclopedia of Genes and Genomes
CNCB: China National Center for Bioinformation
DAVID: Database for Annotation, Visualization and Integrated Discovery
AUC: Area under the curve
ELISA: Enzyme-linked immunosorbent assay
PBS: Phosphate buffer solution
GAPDH: Glyceraldehyde-phosphate dehydrogenase
DHT: Dihydrotestosterone
S.E.M.: Standard error of mean
