# Supplementary figures and images for: Single-cell transcriptome reveals core cell populations and androgen-RXFP2 axis involved in deer antler full regeneration
Source: Cell Regen. 2022 Dec 21;11:43. doi: 10.1186/s13619-022-00153-4 (PMC9772379; doi:10.1186/s13619-022-00153-4)

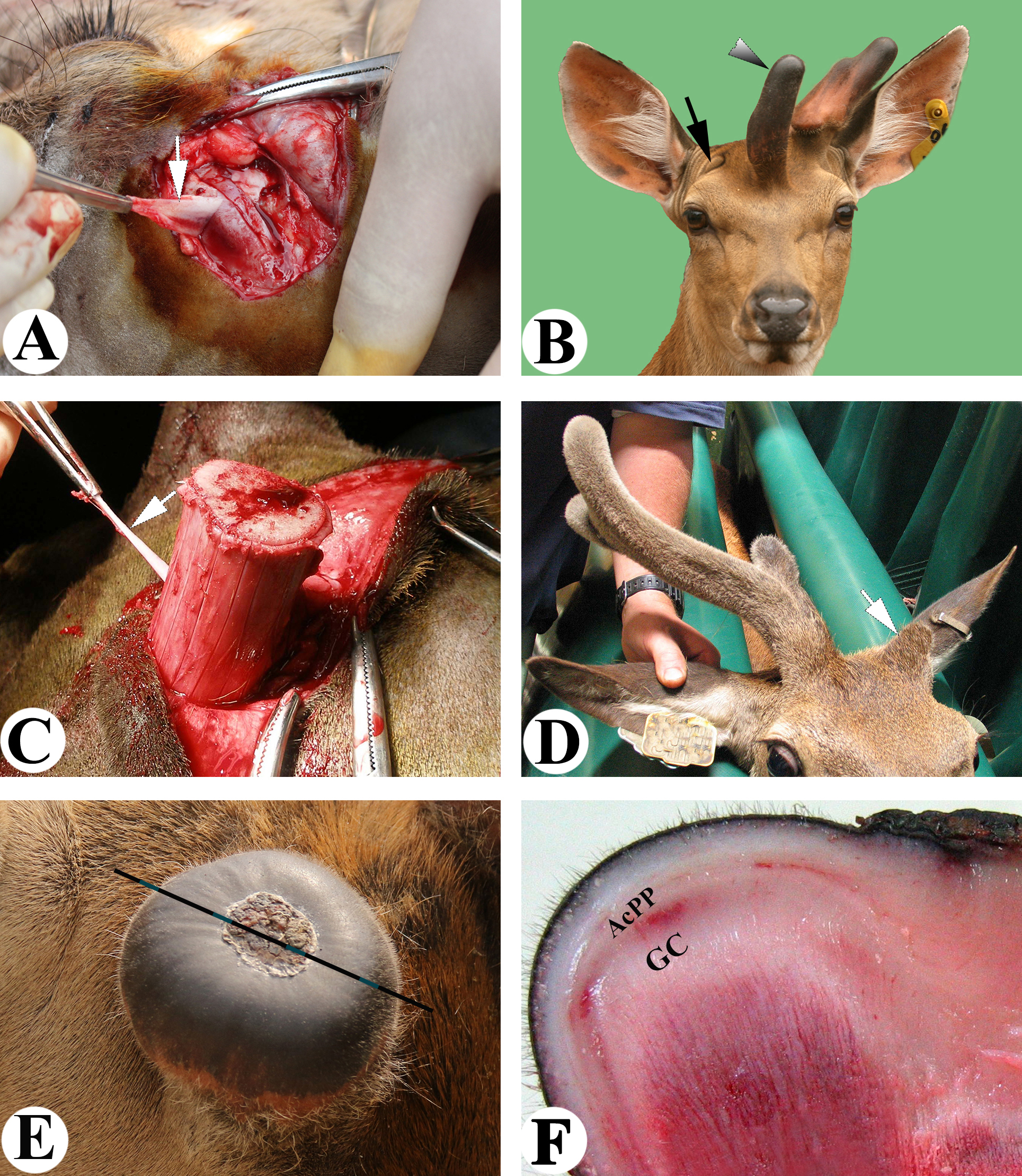

Supplement: Supplementary file 1 — Additional file 1: FigureS1. Antler tissue types and their roles in antler development. (A) Antlerogenic periosteum (AP; arrow).(B) Ectopic antler (arrowhead) formed from the transplanted AP, whereas the presumptive region failed to develop pedicle and antler after missing the AP (arrow); (C) pedicle periosteum (PP; arrow). (D) PP-less pedicle failed to regenerate an antler (arrow), although the contralateral side intact pedicle gave rise to a 3-branched-antler. (E) A fully formed antler blastema (blackline: marked for the longitudinal cutting). (F) Longitudinally cut surface of the antler blastema (AcPP, activated PP tissue; GC, blastema growth center). [file 13619_2022_153_MOESM1_ESM.jpg]

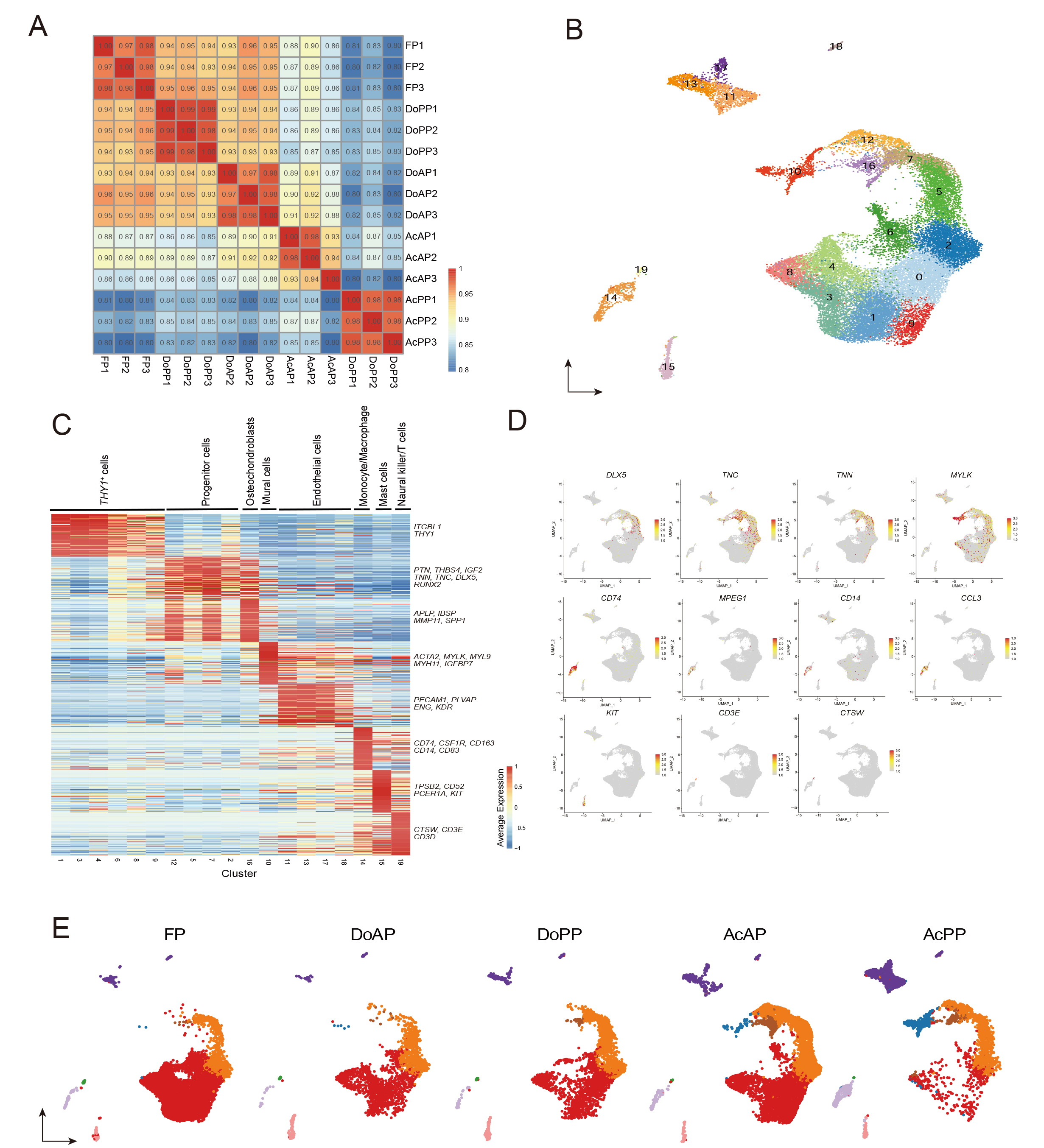

Supplement: Supplementary file 2 — Additional file 2: Figure S2. (A) Pearson correlation coefficients between different tissue samples based on bulk RNA-seq (triplicates of each tissue type). (B) UMAP plot to show the cell clusters that were labeled by both color and number. (C) Heatmap plot to show the top DEGs of each cell cluster. (D) UMAP plots to show the expression patterns of partial marker genes in each cell type (also refer to Figure 1D). (E) UMAP plots to visualize eight cell types in eachof the five tissue types; the color regime for each cell type is the same to Figure1C. [file 13619_2022_153_MOESM2_ESM.jpg]

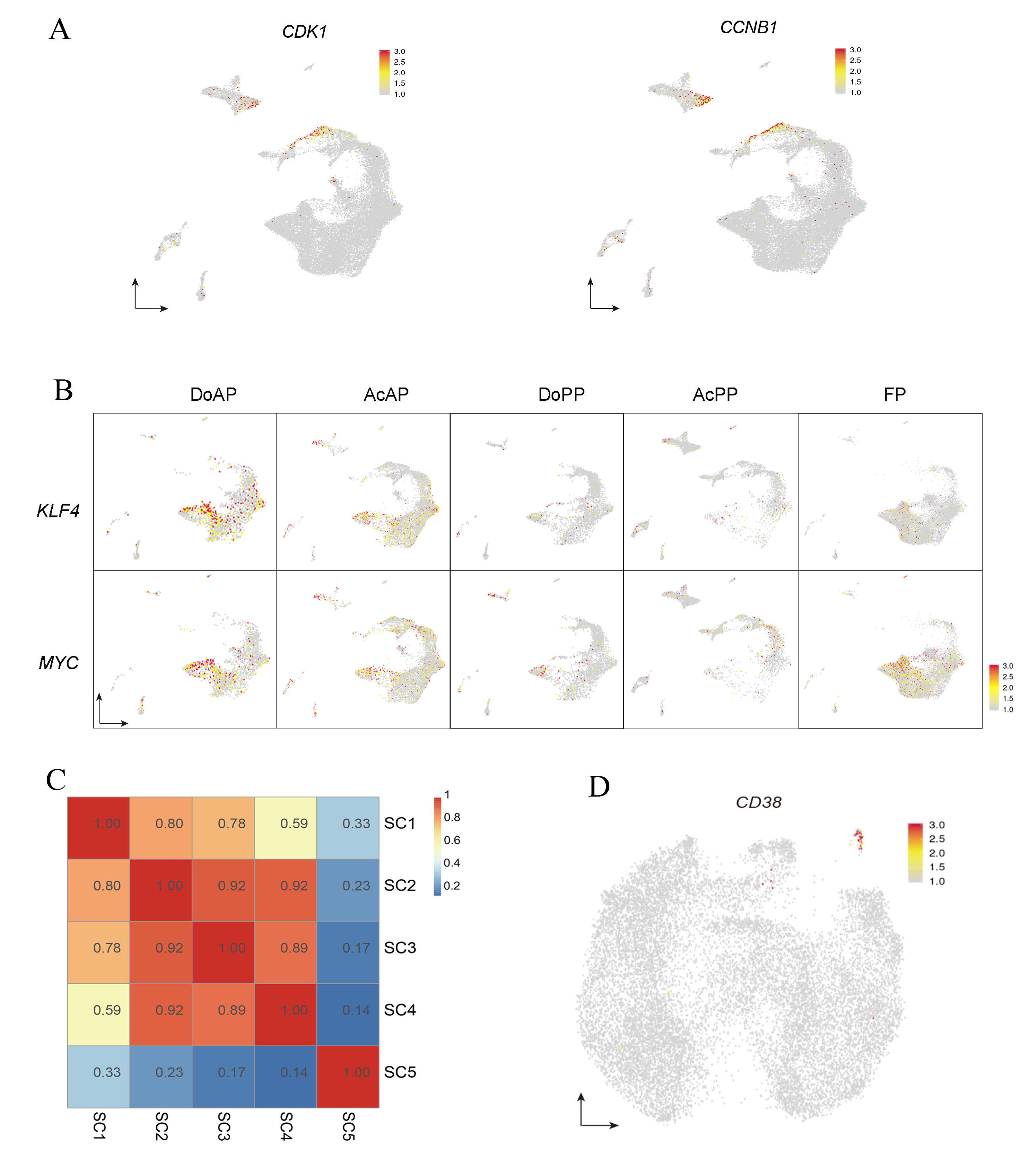

Supplement: Supplementary file 3 — Additional file 3: Figure S3. (A) UMAP plots to visualize the expression patterns of G2-phase specific marker genes, CDK1 and CCNB1. (B) UMAP plots to visualize the expression patterns of marker genes (KLF4 and MYC) in the cells of each of five tissue types. (C) Pearson correlation coefficients between different THY1+cell subclusters. (D) UMAP plot to visualize the expression pattern of immune-related marker gene (CD38) in THY1+cell subclusters. [file 13619_2022_153_MOESM3_ESM.jpg]

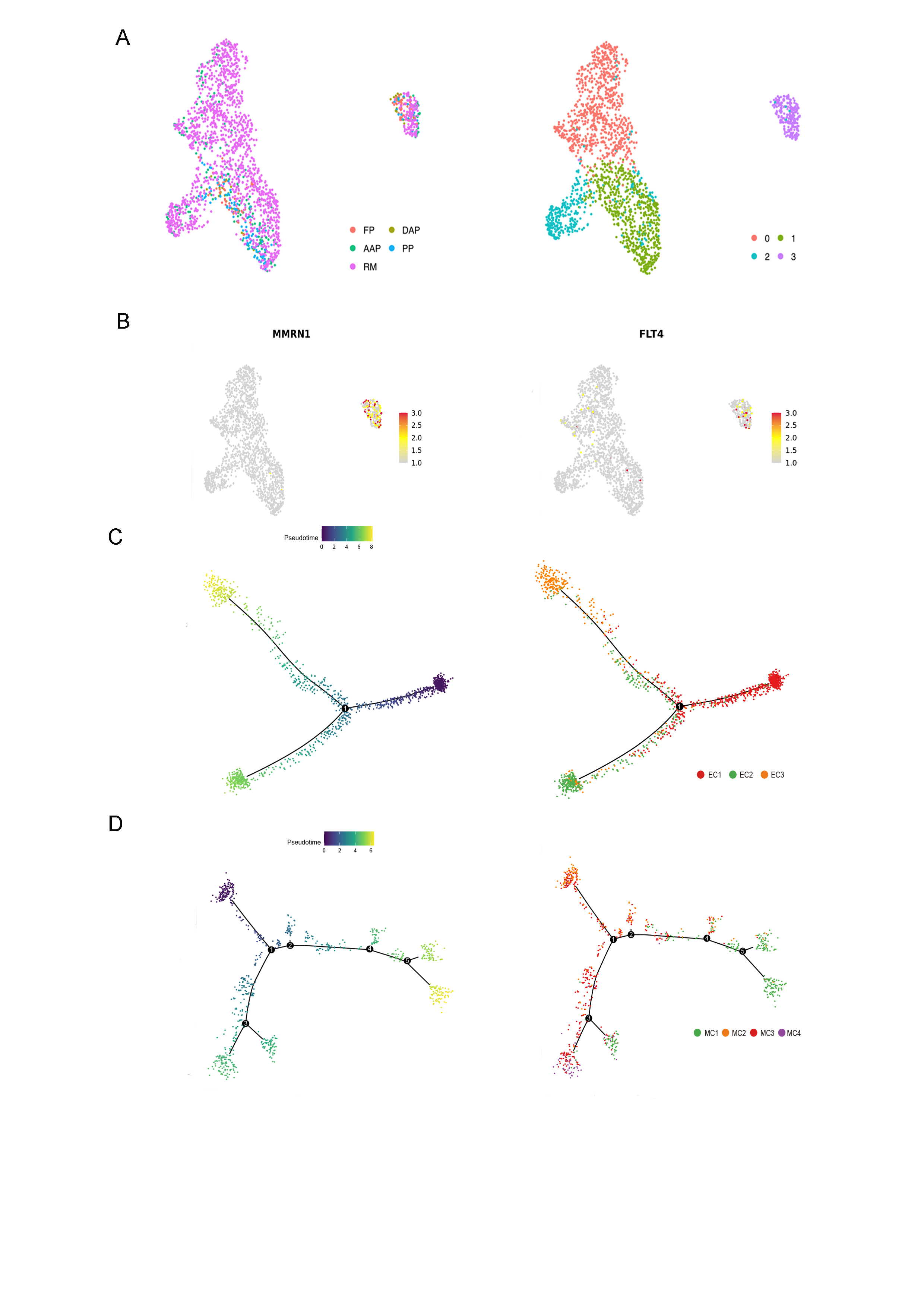

Supplement: Supplementary file 4 — Additional file 4: Figure S4. (A) UMAP plot to visualize the distribution of endothelial cells in five tissue types (left) and in four cell subclusters (right). (B) UMAP plots to visualize the expression pattern of lymphatic marker genes (MMRN1and FLT4). (C) Pseudotime trajectory of three subclusters of endothelial cells. (D) Pseudotime trajectory of four subclustersof mural cells. [file 13619_2022_153_MOESM4_ESM.jpg]

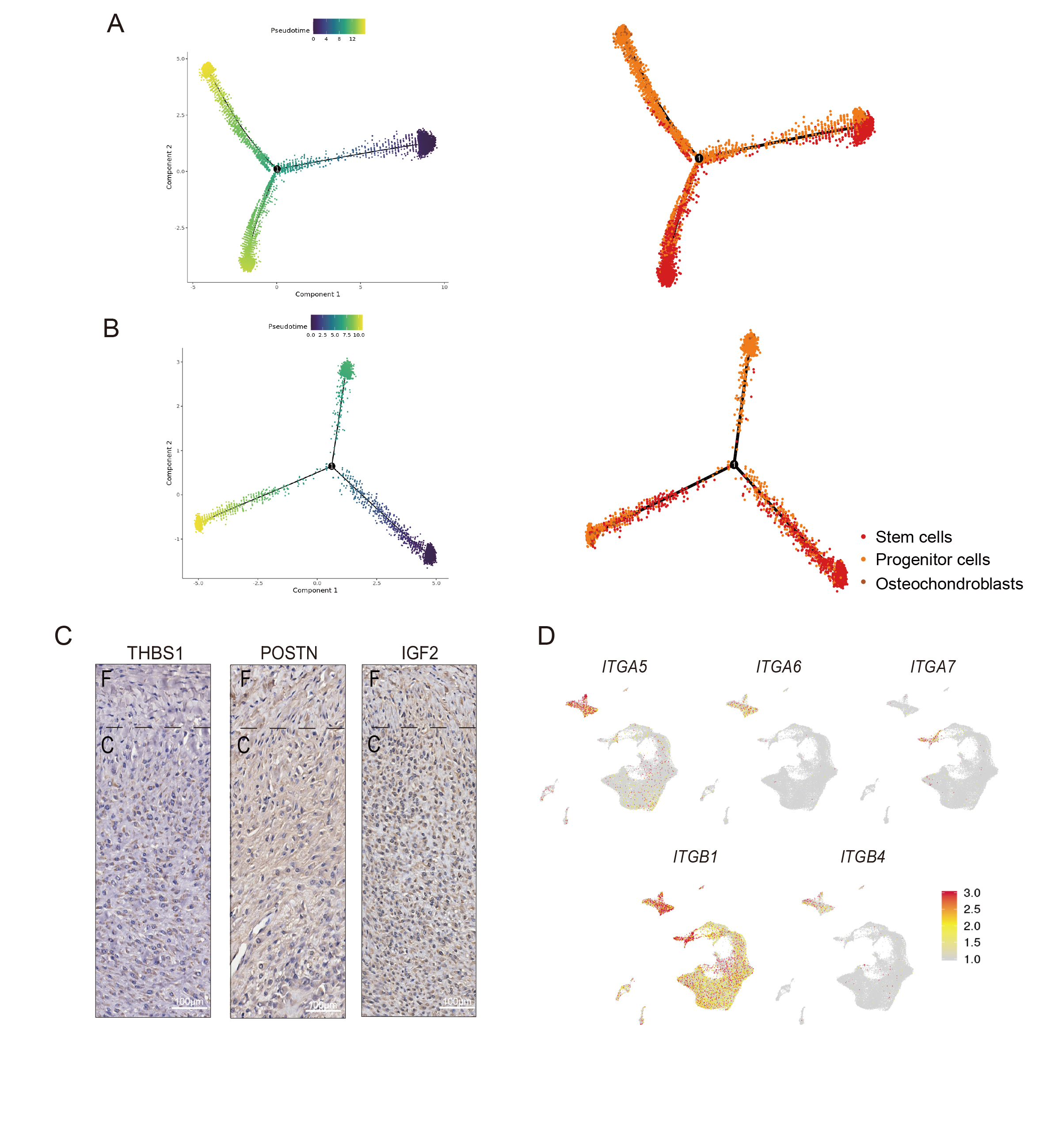

Supplement: Supplementary file 5 — Additional file 5: Figure S5. Pseudotime trajectories of THY1+ cell differentiation in the DoAP and AcAP (A) and that in the DoPP and AcPP (B). (C) IHC staining of THBS1, POSTN and IGF2 in the AcAP. Note that these three factors were found to be highly expressed in the THY1+cells/progenitor cells. Positive staining of POSTN was mainly detected in the extracellular matrix. F, fibrous layer; C, cellular layer. (D) UMAP plots to show the expression pattern of genes, ITGA5, ITGA6, ITGA7, ITGB1, and ITGB4. [file 13619_2022_153_MOESM5_ESM.jpg]

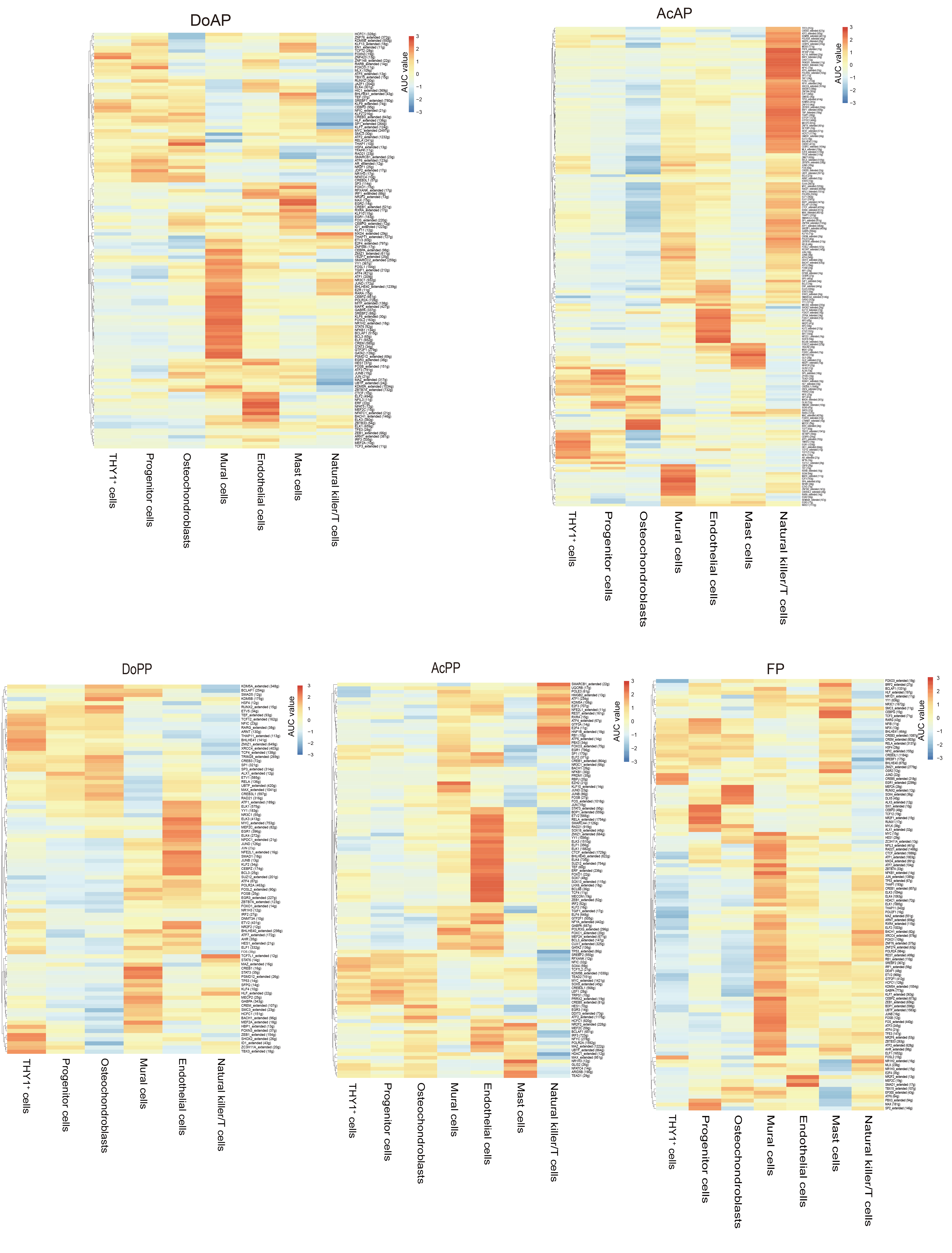

Supplement: Supplementary file 6 — Additional file 6: Figure S6. Heatmap plots to show the regulon activities of the five tissue types. [file 13619_2022_153_MOESM6_ESM.jpg]

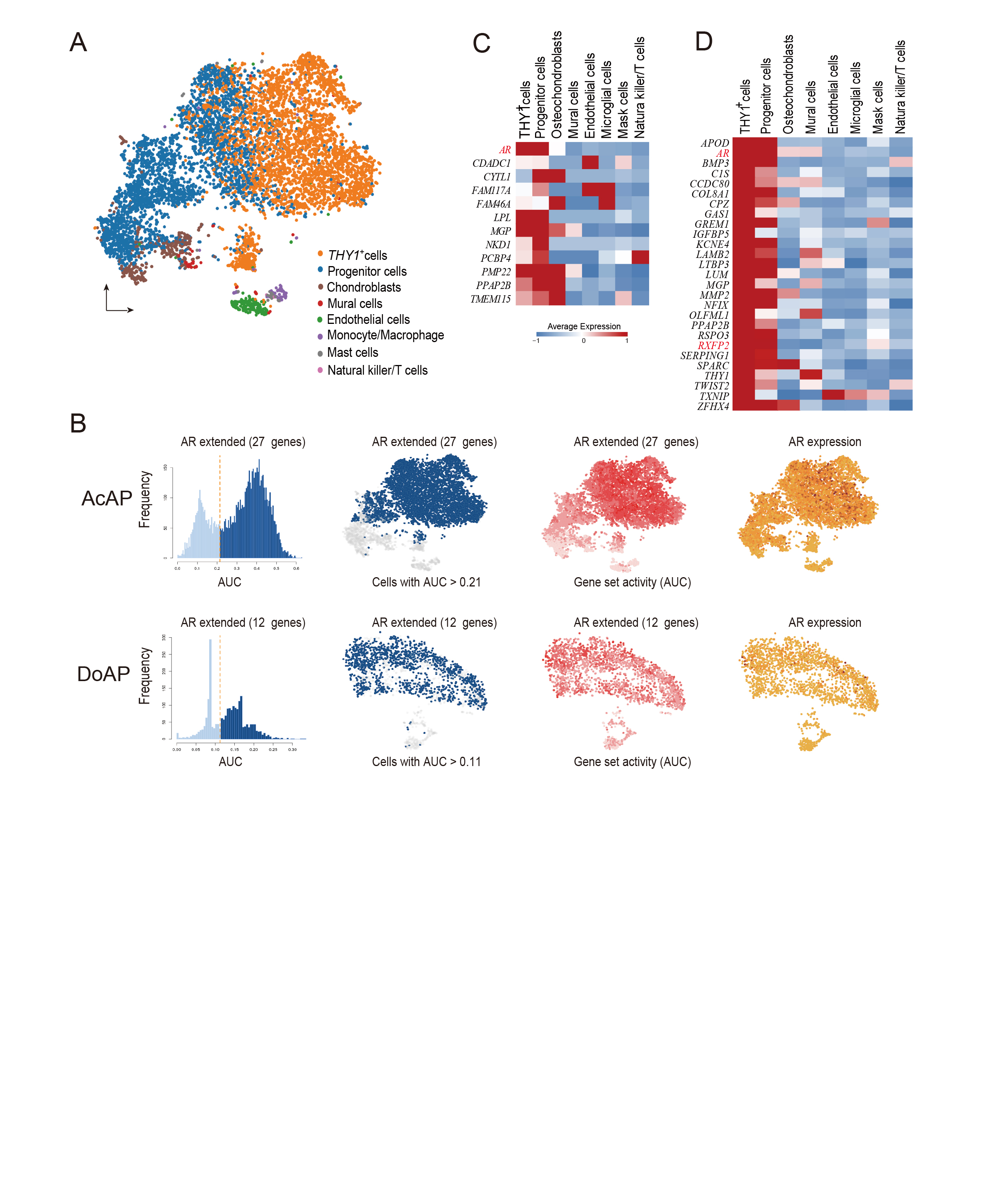

Supplement: Supplementary file 7 — Additional file 7: Figure S7. (A) UMAP plot to visualize the cell composition in the AcAP based on the activity of identified regulons. (B) Activation and expression profile of AR regulon in the AcAP (upper panel) and the DoAP (lower panel). Note that AR regulon in the AcAP (27 genes) was more active than that in the DoAP (12 genes). Heatmap plot to show the average expression levels of genes in AR regulon in the DoAP (C) and AcAP (D). RXFP2 and AR are in red. [file 13619_2022_153_MOESM7_ESM.jpg]
